# Changes in the Distribution of Red Foxes (*Vulpes vulpes*) in Urban Areas in Great Britain: Findings and Limitations of a Media-Driven Nationwide Survey

**DOI:** 10.1371/journal.pone.0099059

**Published:** 2014-06-11

**Authors:** Dawn M. Scott, Maureen J. Berg, Bryony A. Tolhurst, Alienor L. M. Chauvenet, Graham C. Smith, Kelly Neaves, Jamie Lochhead, Philip J. Baker

**Affiliations:** 1 Biology and Biomedical Sciences Division, University of Brighton, Brighton, East Sussex, United Kingdom; 2 National Wildlife Management Centre, Animal Health and Veterinary Laboratories Agency, York, Yorkshire, United Kingdom; 3 Windfall Films, London, United Kingdom; 4 School of Biological Sciences, University of Reading, Reading, Berkshire, United Kingdom; University of Kent, United Kingdom

## Abstract

Urbanization is one of the major forms of habitat alteration occurring at the present time. Although this is typically deleterious to biodiversity, some species flourish within these human-modified landscapes, potentially leading to negative and/or positive interactions between people and wildlife. Hence, up-to-date assessment of urban wildlife populations is important for developing appropriate management strategies. Surveying urban wildlife is limited by land partition and private ownership, rendering many common survey techniques difficult. Garnering public involvement is one solution, but this method is constrained by the inherent biases of non-standardised survey effort associated with voluntary participation. We used a television-led media approach to solicit national participation in an online sightings survey to investigate changes in the distribution of urban foxes in Great Britain and to explore relationships between urban features and fox occurrence and sightings density. Our results show that media-based approaches can generate a large national database on the current distribution of a recognisable species. Fox distribution in England and Wales has changed markedly within the last 25 years, with sightings submitted from 91% of urban areas previously predicted to support few or no foxes. Data were highly skewed with 90% of urban areas having <30 fox sightings per 1000 people km^−2^. The extent of total urban area was the only variable with a significant impact on both fox occurrence and sightings density in urban areas; longitude and percentage of public green urban space were respectively, significantly positively and negatively associated with sightings density only. Latitude, and distance to nearest neighbouring conurbation had no impact on either occurrence or sightings density. Given the limitations associated with this method, further investigations are needed to determine the association between sightings density and actual fox density, and variability of fox density within and between urban areas in Britain.

## Introduction

The world is becoming increasingly urbanized to accommodate the growing human population [1]. In general terms, urbanization leads to the fragmentation and degradation of natural habitats, typically resulting in negative effects on biodiversity [2] [3] [4] and ecosystem services [5]. However, some species can benefit from urbanization by exploiting new habitats and the resources therein [6] [7]. Carnivores considered to be ‘urban exploiters’ are typically medium-sized, dietary generalists that have high reproductive potential and are behaviourally flexible [8] [7]. Such species benefit from the increased availability of anthropogenic food sources and refugia [9], and can reach higher population densities in urban areas than in rural habitats [7] [10]. Living at high densities in close proximity to one another can, however, affect both humans and carnivores. For example, conflicts can arise as a consequence of noise disturbance and damage to property [3] [11] [12]; actual or perceived injuries to people or companion animals [3] [13] [14]; and concerns regarding the transmission of zoonoses [15] [16] or veterinary diseases [17] [18]. Yet, contact with nature can also have positive benefits for human health and well-being [19] [20] [21] and, in many parts of the world, urban residents seek to engage with the wildlife around them, for example, by providing food for garden visitors [9] [22]. Urban wildlife also provide ecosystem services and maintain ecological processes [7] [23]. Timely assessment of urban wildlife populations is thus important for monitoring their benefits and for conflict mitigation and resolution.

The red fox (*Vulpes vulpes*) is globally the most widespread wild terrestrial carnivore [24]. In the last century, this species has colonized urban areas within Europe, Australia, the USA, Canada and Japan [6] [10] [15] [25] [26]. It is found throughout mainland rural Britain and was first reported to be resident in urban areas in southern England during the 1930s [27]. Historically, there has been a strong latitudinal and longitudinal bias in the occurrence of foxes in English towns and cities, being more common in southern regions and rarer in northern and eastern urban areas [28] [29], most likely influenced by the availability of suitable suburban habitats [30].

Urban fox densities typically range from 2–12 adults km^−2^ [31], but can occasionally exceed 30 km^−2^ [10]. In the mid-1990s, the pre-breeding urban population in Britain was estimated as 33,000 individuals [30] [27]. Since the mid-1990s, however, there has been mounting evidence that urban fox numbers and distribution are likely to have changed including: localized increases in density in response to anthropogenic feeding [10] [32]; an outbreak of sarcoptic mange (*Sarcoptes scabiei*) that reduced fox density by >95% in some areas [10] [33]; the colonization of some cities and towns, including some previously considered unsuitable for foxes [34]; and the general expansion and changing structure of urban areas in response to a growing human population and changes in its socioeconomic profile [3] [35]. During this period there have also been several widely-publicized incidents of foxes allegedly biting people [36] and other incidents of conflict, which have led to calls for the implementation of management, as well as increased scientific and public interest in identifying changes in urban fox populations over the course of the last 25 years. In addition, because of their potential role as a vector in disease transmission, monitoring their geographic spread and abundance remains of paramount importance [37].

Quantifying the density and distribution of urban wildlife does, however, pose a number of logistical problems, principally because most land is privately-owned making it difficult to use conventional survey techniques. In contrast, the presence of humans at survey locations lends itself to the use of questionnaire surveys and “citizen science” based approaches have been used successfully to monitor urban wildlife [38]. Questionnaire surveys can be a powerful research tool for quantifying species' distributions across large geographic scales within urban [3], [39], [40] and other [41], [42] habitats [43]. Historically, participants have most frequently been recruited using postal surveys [43], but these can be labour-intensive and expensive [44]. In the modern era, web-based questionnaires potentially represent a more efficient approach for conducting large-scale surveys with significant logistical and cost advantages [45] [46]. However, they are potentially associated with some of the limitations evident in other approaches (e.g., non-random recruitment of participants, reliability of information supplied [43]) in addition to other issues [45]. For example, it is often difficult to target residents in specific areas, such that obtaining information from a representative random sample of people across the entire geographical range of a species can be problematic [47]. Despite this, participant recruitment and geographical reach can both be increased via media coverage [48]. Consequently, web-based questionnaires conducted in conjunction with national media could be an efficient way to obtain current data on a species' distribution over a wide geographic area.

In this study, we assessed the current distribution of urban red foxes in Great Britain (GB) using sightings submitted by the general public via a web-based questionnaire associated with a national television program. These data were used to identify changes in the geographic spread of foxes in urban areas and to re-assess the validity of predictive distribution models produced 25 years ago [29]–[31], [49]. We also explored the relationship between urban features and fox occurrence and sightings density. Specifically we tested whether occurrence and sightings density were (1) lower in northern urban areas [34]; (2) higher in larger urban areas as these are more likely to contain larger suburban zones (i.e. houses and adjacent gardens that are preferred by this species) [31]; (3) lower in urban areas with a higher percentage of public green space as these areas are not typically favoured by foxes because of the lack of secure refugia and high levels of disturbance; and (4) higher in urban areas with closer neighbouring cities, since isolated conurbations may experience lower rates of dispersal from existing populations. We also investigated whether the density of fox sightings reflected expected patterns in the relative density of urban fox populations based upon predictive models developed in the 1980s [29]. Finally, we discuss the limitations of this media-based citizen science approach for surveying urban fox populations.

## Methods

### Ethical Approval

The project was approved by The Pharmacy and Biomedical Sciences School Ethics Committee of The University of Brighton on 23^rd^ April 2012 (Approval code: 1138). Project entitled: ‘Attitudes towards foxes in an urban environment’; investigator: Dr. Dawn Scott. Sightings data were submitted anonymously and publicly volunteered.

### The study area

Great Britain consists of England, Wales and Scotland. It covers 229,848 km^2^ of which 6.3% (14,546 km^2^) was classified as urban based on the 2001 urban boundaries of conurbations containing ≥20,000 people [50]. The UK (GB and Northern Ireland) human population is projected to increase from 62.3 million in 2010 to 67.2 million by 2020; of these, >80% will live in urban areas [51].

### Data collection and participant recruitment

Between 29^th^ April and 14^th^ May 2012, the British public were asked to submit the date, time and location of any direct visual sightings of red foxes in urban areas during 2012 via a website produced in association with a nationwide series of television programs called “Foxes live: wild in the city” on Channel 4, one of the major terrestrial broadcasters. Accompanying images could be submitted with sightings records. The first one-hour program was broadcast on the 29^th^ April 2012 at 8pm. This was followed by a series of six 1-minute programs in the following week at approximately 6pm, concluding with three one-hour programmes at 8pm on 7^th^, 8^th^ and 9^th^ May. Online submission via the website (http://foxes.channel4.com/) closed on midnight of the 14^th^ May 2012. An interactive link with *Google maps* enabled accurate geo-referencing of submitted information. Sightings were checked for errors in date and location and any obviously erroneous data (e.g., located off-shore) were removed prior to analysis. As our focus was on foxes in urban areas, any sightings originating from non-urban locations were also excluded.

### Patterns in urban fox distribution and density

Urban fox sightings were mapped using Arc GIS 10 [52]. Initial urban boundaries were defined using the most up-to-date urban boundary data set (Census 2001 Urban Areas) [50]; urban areas were defined as conurbations with populations of ≥20,000 people. These boundaries were used to identify the broad-scale distribution of foxes in urban areas across Britain.

However, since the frequency of sightings needs to be corrected for human density, a second more precise urban layer was created using a more restricted definition of urban extent and which focussed only on England and Wales to increase comparability with previous analyses [30]. A map of administrative urban areas was created using the lower layer super output area 2011 shapefiles from the Office of National Statistics (ONS) [51], the mid-2010 ONS population survey [53] and the 2007 Land Cover map (LCM 2007 [54]). Polygons in which the total human density was ≥1000 people km^−2^ were identified and conurbations defined as groups of these contiguous polygons. Conurbations with <30,000 people were removed. The 1 km resolution land cover raster layer (LCM 2007) was then superimposed onto the map of administrative urban areas and cells not classified as urban or suburban (land cover classes 22 and 23) were removed. A grid with a 2×2 km resolution was then applied using the fishnet tool in Arc GIS 10: grid cells were retained if the centre of the 4 km^2^ area fell within an administrative urban area. This resulted in 144 towns and cities (hereafter “conurbations”) across England and Wales (see Table S1).

Total urban area (km^2^), total human population, human density, fox sightings density, latitude, longitude and Euclidean distance to nearest neighbour (calculated from the midpoint of each conurbation) were calculated for each town and city. The three latter measures were calculated based on the recorded location of cities in the OS OpenData Stategi settlement data set (www.ordnancesurvey.co.uk). To estimate the proportion of semi-natural habitats in each conurbation, our map of urban areas was superimposed onto one created by Richardson and Mitchell (2010) which map used the Generalised Land Use Database (GLUD; England) and the Coordination of Information on the Environment (CORINE; England and Wales) to determine the area of green space (>5 m^2^ excluding residential gardens) in conurbations calculated at the level of electoral wards [55]. The percentage of green space in wards that intersected each conurbation was then calculated. Observed fox sightings density was corrected for “survey effort” by dividing by human density (hereafter “sightings density”).

To quantify changes in urban fox distribution over the last 25 years, and to determine if sightings density concurred with predictive models developed in the 1980s, we compared our data with those of Harris and Smith (1987) [30]. In particular, we: (a) calculated the proportion of cities (n = 65) that were deemed to have zero or very low fox densities in the 1980s [30], but which had sightings submitted in 2012; and examined whether there was a correlation between current sightings density and (b) mean fox density (family groups km^−2^) as determined in 14 ‘predictor’ cities by Harris and Smith (1987) and (c) predicted mean fox density in a subset of 55 cities estimated using a habitat suitability model developed by these authors [30]. In addition, 2012 data recorded for Scotland were compared to data collected during a 2006 local authority questionnaire survey of Scottish cities [56].

The impact of latitude, longitude, extent of (log-transformed) urban area, distance to nearest neighbouring city, the percentage of green space, and the interaction between extent of urban area and percentage of green space on sightings density were investigated using multivariate models in R (v3.0.2) [57]. Sightings density was distributed as a zero-inflated continuous variable, a type of distribution for which there is no standard analytical approach and for which transformation is unlikely to normalise the data. Therefore, we followed a two-step procedure similar to that for analysing zero-inflated count data. Firstly, the sightings data were converted to a binomial response variable (foxes present or absent) and investigated the probability of fox presence using a generalised linear model with a binomial error structure. Secondly, conurbations with a sightings density of zero were removed and the remaining data normalised using a log-transformation: linear regression was then used to investigate factors affecting sightings density given that it was greater than zero. We initially investigated the presence of spatial autocorrelation in both the binary variable (presence/absence) and the sightings density variable using permutation tests for Moran's I and Geary C's statistics (package spdep in R [58]). This involved creating a connectivity matrix between each point of the datasets. Several maximum distances between connected points were tested (i.e. 1, 5 and 10 km) and the most appropriate connectivity matrix was identified. The matrix was then weighted based on spatial interactions [59]. Because we had no *a priori* knowledge of the shape of these interactions, we ran permutation tests using the matrix weighted by all available specifications in the package spdep (1000 simulations). If spatial autocorrelation was evident in the data, Moran eigenvectors that model positive correlation (MEM method; see [60]) were used as covariates in the regressions [59].

For both statistical procedures, Akaike's Information Criterion for small datasets (AICc) [61] was used to rank models and create a set of candidate models that best explained the data: we considered models that contributed to 95% of the cumulative weight as plausible and used model averaging across the set to estimate parameter values [58]. To assess model fit, we used goodness-of-fit testing and calculation of over-dispersion metrics for the generalised linear models and inspected patterns of the residuals for linear models. Predictor variables that were found to be important in both the binomial regression and the linear regression were considered potentially important for explaining patterns of occurrence.

## Results

Viewing figures for the first television broadcast were estimated as 1.9 million (Channel 4 pers. comm.), equivalent to 3.0% of the British population [53]. Overall, 17,477 sightings were submitted during the 15-day period: 55 records were excluded due to errors in date and/or location; 15,705 records were from urban areas and, of these, 2808 (17.9%) had photos or images of foxes attached. Sightings spanned the period January 18^th^ to May 14^th^ 2012 inclusive, with 47.0% of sightings occurring between 19:00BST and midnight.

### Patterns in urban fox distribution

**Figure 1:**
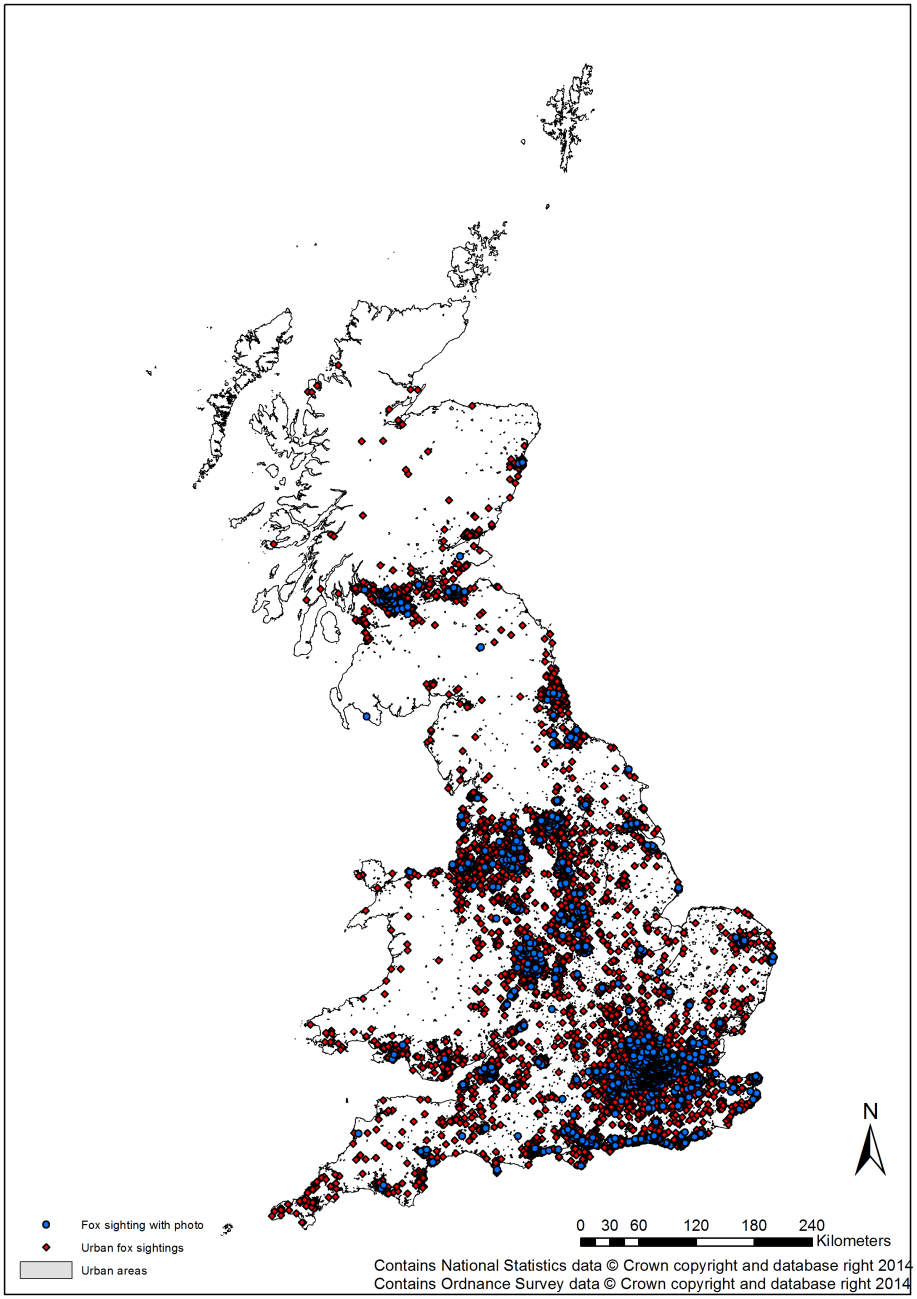
Current distribution of urban foxes in Great Britain. Current distribution of urban foxes in Britain based upon sightings reported in urban areas in response to a national television programme in 2012. Red dots indicate sightings; blue dots indicate sightings with supporting photographic evidence.

**Figure 2:**
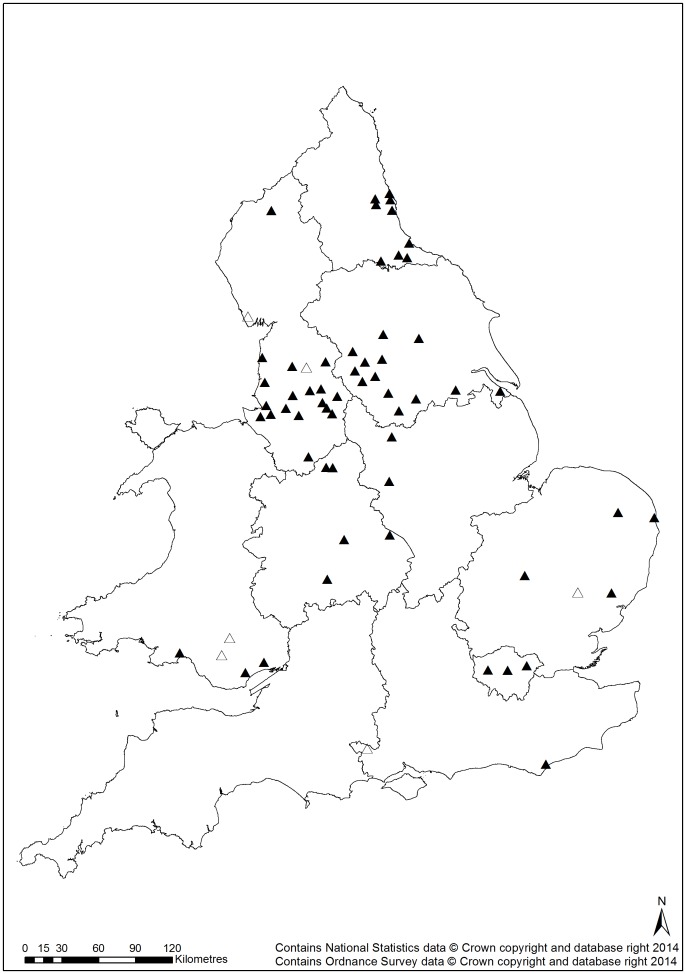
Changes in fox presence since 1987. Records of fox presence in the 65 cities predicted by Smith & Harris (1987) to have zero or low fox densities. Closed triangles indicate cities where foxes were sighted in 2012; open triangles indicate cities where no fox sightings were reported in 2012 survey.

All English and Scottish conurbations previously reported to have urban fox populations [30] [34] were identified in the current survey i.e. no established fox populations had disappeared (Figure 1). However, it is clear that the geographic extent of foxes in urban areas has increased since 1987 and further since 2001, with foxes sighted in many northern and eastern conurbations where they had previously been absent (Figure 2). Of the 65 cities reported to have few or no foxes present in 1987 59 (90.8%) had sightings submitted in 2012. Consequently, there is now little apparent longitudinal or latitudinal pattern in occurrence. Sightings in Scottish towns and cities were similar to those reported in 2006 [56].

**Figure 3:**
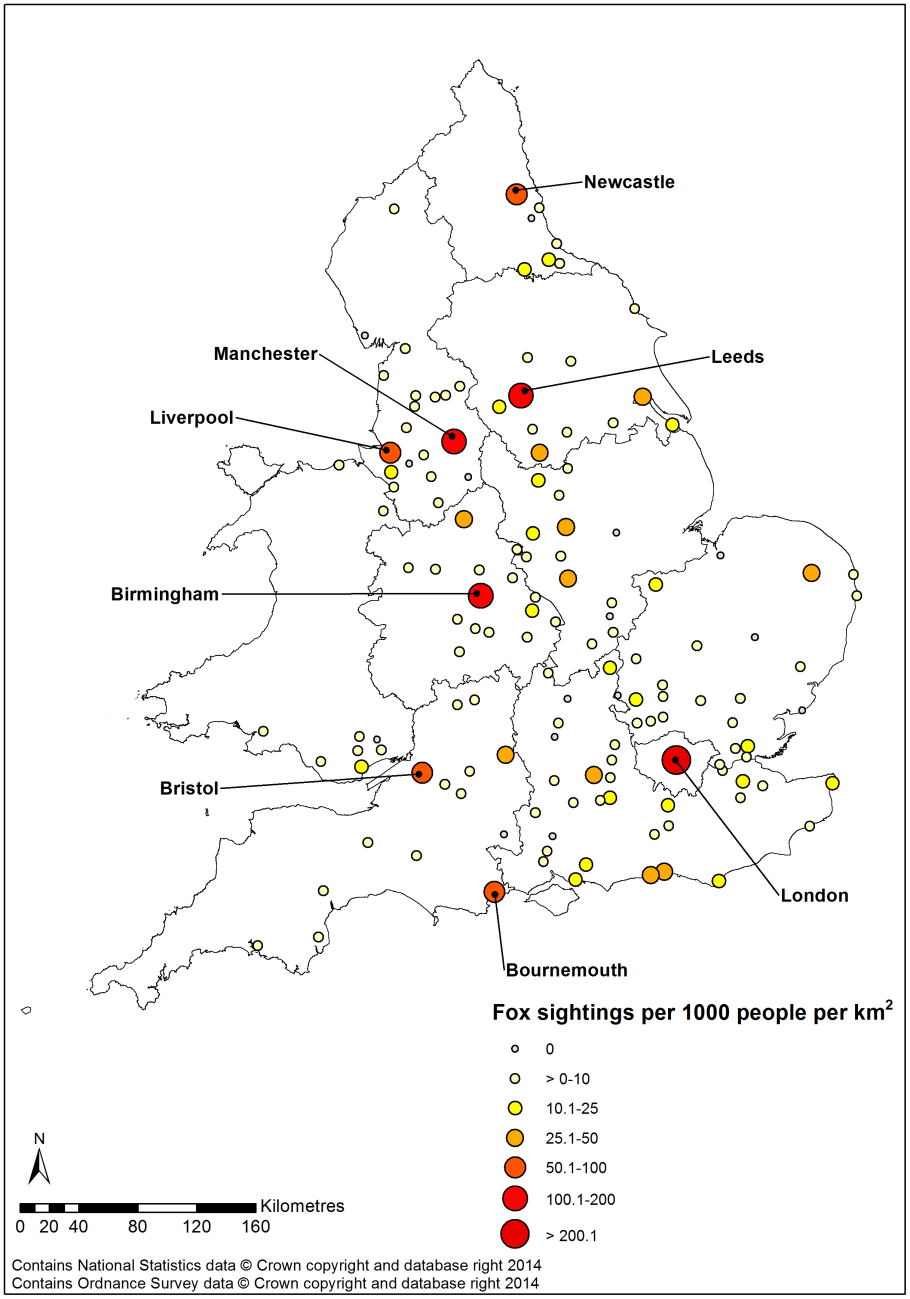
Density of fox sightings in Great Britain. Density of fox sightings based on publicity submitted sightings in the 144 cities used within statistical models. The size of circles depicts the number of fox sightings per 1000^2^.

The majority of conurbations had a low sightings density (Figure 3): 40.3% had <2 fox sightings per 1000 people km^−2^ and 90.3% had <30 fox sightings per 1000 people km^−2^. Due to recent amalgamations of urban areas (Birmingham, Dudley, Solihull, Walsall and Wolverhampton), only eight of the 14 predictor cities in [30] were directly comparable to the conurbations identified in this study (Bath, Bournemouth/Poole, Bristol, Cheltenham, Coventry, Gloucester, Leicester, Nottingham). Based on these eight locations, there was no significant correlation between mean fox density (family groups km^−2^) [30] and sightings density (Pearson's correlation: r = 0.302, p = 0.430).

Similarly, directly comparable data were only available for 15 cities where mean fox density had been estimated by Harris and Smith (1987) [30]. Predicted mean fox density was not significantly correlated with sightings density (Pearson's correlation: r = 0.142, p = 0.615).

### Probability of fox presence in conurbations

We found no evidence of spatial autocorrelation in the binary variable (n = 144; optimal neighbouring distance 1 km); for both permutation tests and all weighting specifications, p-values were >0.5. We therefore did not need to account for spatial autocorrelation in further analyses of presence/absence data.

Twelve models plausibly fitted the presence/absence data and all possible covariates appeared at least once in the candidate set. For each plausible model, the dispersion parameter was <1.06 indicating little over-dispersion in the data. Moreover, no candidate models showed evidence of a poor fit using the un-weighted sums of squares (all P-values >0.31). We thus proceeded with model averaging across the 12 models (Table 1). The only covariate for which 95% confidence intervals did not include zero was the extent of the urban area: foxes were more likely to have been reported in larger cities.

**Table 1:** Model-averaged estimates of explanatory variables in models examining the presence/absence of fox sightings.

Parameter	Mean	S.E.	95% C.I.	Relative Importance
Intercept	7.82	33.92	[−58.67–74.31]	-
Latitude	−0.84	0.51	[−1.85–0.16]	0.53
Longitude	0.09	0.36	[−0.61–0.79]	0.20
**Log (Urban extent)**	**5.49**	**2.13**	**[1.32–9.66]**	**1.00**
Distance to neighbour	−0.03	0.04	[−0.1–0.05]	0.33
% green space	−0.04	0.06	[−0.15–0.07]	0.37
% green space * log (urban extent)	−0.25	0.25	[−0.75–0.25]	0.12

Model-averaged estimates for the explanatory variables present in the 11 top models of probability of fox presence. The relative importance is the sum of the AIC weight over all models including the explanatory variable. Significant variables are shown in bold.

### Correlates of sightings density

Similarly, there was no evidence of spatial autocorrelation in the non-zero sightings data (n = 129; optimal neighbouring distance 1 km); for both permutation tests and all weighting specifications, p-values were >0.7. Consequently, there was no need to account for spatial autocorrelation in further analyses of these data.

Six candidate models were identified, which contained each explanatory variable at least once. Visual examination of plots of model residuals versus both fitted and leverage values showed very little pattern, indicating that they were a good fit to the data. Model averaged values indicated that urban area (on the log scale), longitude and percentage of public green space all had a 95% confidence interval that did not include zero (Table 2): urban extent and longitude were positively related, while percentage of public green space was negatively related, to non-zero sightings density.

**Table 2:** Model-averaged estimates of explanatory variables in models examining corrected density of fox sightings for those conurbations where ≥1 sightings were submitted.

Parameter	Mean	S.E.	95% C.I.	Relative Importance
Intercept	−1.85	4.41	[−10.5–6.8]	-
Latitude	−0.13	0.07	[−0.27–0.01]	0.81
**Longitude**	**0.22**	**0.06**	**[0.1–0.34]**	**1.00**
**Log (Urban extent)**	**1.25**	**0.09**	**[1.07–1.43]**	**1.00**
Distance to neighbour	−0.002	0.007	[−0.02–0.01]	0.29
**% green space**	**−0.03**	**0.01**	**[−0.05– −0.01]**	**1.00**
% green space * log (urban extent)	0.01	0.01	[−0.01–0.02]	0.39

Model-averaged estimates for the explanatory variables present in the six top models of corrected fox sightings for those conurbations where at least one sighting was submitted. S.E. stands for standard error and C.I. for confidence interval. The relative importance is the sum of the AIC weight over all models including the explanatory variable. Significant variables are shown in bold.

## Discussion

### Colonisation of conurbations by foxes

As evident from earlier studies [34] [40], the distribution of urban foxes in Great Britain appears to have changed markedly in the last 25 years. For example, sightings were recorded from 91% of 65 cities where they had previously been considered rare or absent in an earlier study in the 1980s [30]. Consequently, the historic north/south divide in the presence of foxes in urban areas is now less pronounced. The reasons associated with this expansion are, however, unclear.

Although several studies have considered the factors associated with contemporary colonisations of urban habitats by birds (e.g., [62], [63]), there are comparatively few data for mammals [64] [7]. Colonisation is likely to be a multi-faceted process involving, at the most basic level, the availability of suitable habitats within the new location(s) and a source population of individuals capable of moving into those areas.

Although some public green spaces, such as woodlands, may be favoured by low-density populations [65], established urban fox populations tend to exhibit a strong preference for residential gardens, particularly medium-sized gardens associated with low-density housing constructed in the inter-war years [30]. This is likely to explain the observed negative relationship between sightings density and public green spaces, as residential green spaces would provide greater opportunities for both refugia and food resources in comparison to large open spaces, such as amenity grasslands. Therefore, one possible explanation for the observed changes in occurrence could be that the infrastructure of recently colonized locations has changed to more closely match that present in cities where foxes have been present for longer [31]. Yet the majority of development in Britain in this time period has been associated with changes that are not likely to have been beneficial for foxes e.g., in-filling of existing gardens (“garden grabbing”), the re-use of derelict land and the construction of high-density housing with small gardens [3], [35]. Consequently, substantially more information is required to be able to identify why foxes have undergone a period of rapid colonisation in the last two decades. Of particular help would be identifying the spatio-temporal pattern of colonisation within individual cities in relation to the changes in the associated infrastructure [66], [67].

Source populations for the colonisation of conurbations could be either from the surrounding rural landscape or from foxes dispersing from towns and cities already occupied. Although the colonisation of some European cities has been attributed to a rise in rural fox populations following the successful management of rabies [6], [26], data on patterns of change in rural fox populations in Britain are not entirely clear, partly because of the limitations associated with the monitoring methods currently being used (e.g., “game bags”, faecal counts, road traffic casualties).However, in a review of these data, Battersby (2005) [68] concluded that, at a national level, fox populations appear to have been relatively stable since circa 1995, the time period corresponding to the present study. Therefore, whilst acknowledging their limitations, those data available do not appear to strongly support a hypothesis of rapidly changing rural fox populations as a factor driving the colonisation of new urban areas.

Two mechanisms that might favour colonisation are: (i) if colonising individuals originated from nearby urban conurbations; and/or (ii) if rural individuals were able to access patches of semi-natural habitat within the urban matrix and use these as a stepping stone from which to further colonise the wider urban landscape. We could not, however, find evidence to support either mechanism; neither nearest neighbour distance nor the percentage cover of green space significantly affected the likelihood that a conurbation was occupied *per se*. In part, these non-significant differences could have arisen because foxes are capable of dispersing over large distances [72], such that immigrating individuals could have arisen from more distant conurbations. One further factor that could promote colonisation is the preference for habitats similar to those experienced in an individual's natal range [69] [70], [71] [72]. Distinguishing the origins of colonising individuals, the number of founder events, and the relatedness between rural and urban populations with the use of genetic studies [26] would significantly help to elucidate the colonisation process.

### Limitations of the study

The main limitations of the current study were associated with the approach used to enlist volunteers, the extent of coverage achieved, and the non-standardisation of effort to allow validation of null data. Although media-driven web-based surveys have the potential for collecting a large amount of data at a national scale [38], there are biases associated with the audience reached and hence the suitability of information received [73]. For example, participants in this study would have been limited to the subset of people that actively chose to watch the television program and who were motivated to report sightings of foxes. Moreover, the reliance on a web-based approach further restricted responses to those with access to appropriate technology and the necessary technical skill to submit information correctly; this is not likely to have been a significant issue in this study, since a single fox territory typically contains several hundred households and internet provision and mobile phone coverage in urban areas in Britain is very high. In addition, problems associated with volunteer recruitment can be further minimised by making the information requested as simple as possible, although this in turn limits the amount of detailed information that can be collected [73]. In this regard, there is a compromise between broadcasters and scientists, with final decisions predominantly in the hands of the former. With simplifications in design to engage a wider audience, we have had to restrict analyses to consider relationships at relatively broad spatial scales.

The reliance on sightings of foxes alone is also associated with an absence of negative information. The submission of negative information does, however, pose its own particular problems, since many non-scientists are intrinsically less aware of the usefulness of information relating to the absence of a species. Furthermore, there is then the difficulty of discriminating between true (participants had not observed foxes in the specified time period) and false (people had not submitted a sighting even though they had observed foxes) absences. These can be identified using follow-up “ground-truthing” studies in a sub-set of cities to determine the presence/absence of foxes but perhaps more usefully to quantify the relationship between true densities relative to sightings density. However, these will likely be expensive and time-consuming, factors which would probably be highly influential in influencing decisions to adopt a media-driven approach in the first place. It is recommended, therefore, that researchers should aim to maximise capture of both negative data and survey effort where possible, by emphasizing the need for these data in the recruitment process and survey design. In addition, it is our experience that one media appearance is likely to result in additional invitations which can potentially provide opportunities for some ground-truthing; for example, using this approach we have been able to confirm the presence of foxes in some towns and cities not identified in the primary data set presented here. In addition, with standardisation in effort and methodological approach, longitudinal media-based studies could be implemented to monitor changes in both distribution and relative abundance, although this is likely to be difficult given the different objectives of broadcast media versus scientists.

One further potential problem to consider in citizen science based surveys is the reliability of the information received [45]. In this study, we requested sightings of a distinctive, well-known animal for which there are no morphologically similar species present in Britain (other than some domestic dogs). Furthermore, most sightings are likely to have been made over short distances e.g., within the participant's garden. Therefore, we are confident that those sightings reported were reliable: further validation of the information received was achieved by asking people to submit photographs. All photos were checked and confirmed to be of foxes with no errors in identification, although one was a deliberate erroneous “sighting” as it was a modified image. However, it was not possible to eliminate deliberately false reporting. For example, foxes have previously been associated with a hoax on one offshore island [74] and are the subject of heated debate concerning the need for culling in both rural and urban habitats following a ban on hunting foxes with dogs and alleged attacks on people [36] respectively. Consequently, there is the possibility that some sightings could have been erroneous [45], although reports of foxes in many conurbations were corroborated by at least one sighting accompanied with a photograph (see Figure 1).

In contrast, the data collected are more problematic in the context of elucidating patterns in the relative density of foxes between urban areas and quantifying the distribution of foxes within individual locations. Although coverage in terms of overall viewing figures was very good, with 3.0% of the British population watching the first program, clearly demonstrating the potential of media-based studies in ecological studies, only 17,477 sightings were submitted (<0.1% of the total population). Given that fox densities vary markedly within individual conurbations [31],[75],[76], this low coverage presents considerable challenges for obtaining detailed information about fox ecology at fine spatial scales, particularly in less favourable and less populated habitats within the urban landscape. Therefore, the primary limitation in this study was getting viewers to submit sightings rather than increasing the number of viewers. This is likely to be a significant issue in media-driven studies generally and one that may be hard to resolve, since there is often limited scope for explaining methodological approaches in detail although approaches for incentivising viewers do exist (e.g., corporate sponsors offering prizes).

Perhaps not surprisingly, therefore, with the exception of urban extent, we did not detect any statistically significant relationships between sightings density and the mean density of foxes in cities in the 1980s either for those locations where foxes were surveyed directly or where density was estimated using the resultant habitat suitability model [30]. Although it is fair to assume that these results, at least in part, reflect the limitations of the current methodology (i.e. larger cities are occupied by more potential participants), the absence of any significant relationship would also arise if fox populations within these cities had changed differently relative to one another over the past 25 years. However, with one notable exception [77] [78], there are no up-to-date estimates of fox density in any towns or cities in Britain. Nevertheless the results of this and previous studies suggest that new conurbations have been colonised [34], that some populations have been impacted by sarcoptic mange [33] [32] and that others may have increased locally in response to anthropogenic feeding [32]. Consequently, there is a need to re-examine the habitat suitability model developed by Harris and Smith (1987) [30] to determine whether it still adequately describes the distribution of foxes within and between towns and cities in Britain. Such studies would also benefit from considering total fox density rather than the density of family groups, since long-term studies have indicated that group size and productivity are more variable than previously assumed [32] [77] [78].

## Conclusions

This study has demonstrated that urban foxes are now more widespread within Great Britain than previously recorded, and that many towns and cities previously considered unsuitable now contain fox populations, albeit probably at relatively low densities. The ecological processes underlying these recent colonisation events are unclear and require further investigation, but this altered distribution does pose concerns in the context of the possible introduction and management of zoonotic diseases and parasitic infections [79]–[82]. Therefore, there is a need to revise and extend earlier models for predicting the distribution and densities of urban foxes.

From a methodological perspective, the television programs that formed the basis for collecting the sightings data were very successful in mapping the changing distribution of foxes in towns and cities. However, a number of problems are evident in the context of using these data to investigate fine-scale processes, most notably due to the pattern of coverage associated with participants and because the emphasis on the submission of positive information makes it difficult to identify true and false absences. Consequently, detailed follow-up studies are required to address some of these constraints.

## Supporting Information

Table S1 **144 cities used in multivariate additive models.** The table shows for each conurbation latitude and longitude, extent of urban area (in km^2^), the total population present, the human density, % public green space and the observed fox density. Yellow highlighted cities are those previous used as predictor cities in the 1987 model by Harris and Smith. Grey highlighted are cities previously believed to have few or no foxes present in 1987 and pink highlights are cities with previous predicted mean fox densities estimates from 1987.
(DOCX)

Licence S1 **Digimap licence agreement.**
(DOCX)

Licence S2 **Ordinance Survey (OS) open data licence.**
(PDF)
