# Supplementary material for: Changes in the Distribution of Red Foxes (Vulpes vulpes) in Urban Areas in Great Britain: Findings and Limitations of a Media-Driven Nationwide Survey
Source: PLoS One. 2014 Jun 11;9(6):e99059. doi: 10.1371/journal.pone.0099059 (PMC4053368; doi:10.1371/journal.pone.0099059)
Supplement: Table S1 — 144 cities used in multivariate additive models. The table shows for each conurbation latitude and longitude, extent of urban area (in km2), the total population present, the human density, % public green space and the observed fox density. Yellow highlighted cities are those previous used as predictor cities in the 1987 model by Harris and Smith. Grey highlighted are cities previously believed to have few or no foxes present in 1987 and pink highlights are cities with previous predicted mean fox densities estimates from 1987. (DOCX) [file pone.0099059.s001.docx]

Table S1: 144 cities used in multivariate additive models.

| City or conurbation name | Longitude | Latitude | Area (km^2^) | Total population | Population density per km^2^ | Fox sightings | Fox sightings per person  per km^2^ *1000 | Green space (%) |
| --- | --- | --- | --- | --- | --- | --- | --- | --- |
| ABINGDON | -1.28242 | 51.66876 | 9.1 | 32183 | 3537.2 | 0 | 0.000 | 56.7 |
| ANDOVER | -1.48048 | 51.20655 | 10.5 | 33159 | 3173.6 | 1 | 0.315 | 65.8 |
| BANBURY | -1.33890 | 52.05971 | 11.8 | 40220 | 3417.4 | 3 | 0.878 | 63.4 |
| BARNSLEY | -1.47935 | 53.55195 | 64.8 | 138220 | 2132.9 | 3 | 1.407 | 68.6 |
| BARROW-IN-FURNESS | -3.21725 | 54.12043 | 11.9 | 39976 | 3351.3 | 0 | 0.000 | 49.8 |
| BASILDON | 0.48736 | 51.57292 | 26.1 | 93431 | 3584.2 | 29 | 8.091 | 61.7 |
| BASINGSTOKE | -1.10596 | 51.26485 | 31.6 | 98582 | 3121.8 | 9 | 2.883 | 47.1 |
| BATH^1^ | -2.35925 | 51.3794 | 24.1 | 88756 | 3689.5 | 3 | 0.813 | 49.9 |
| BEDFORD | -0.46571 | 52.13898 | 25.8 | 100528 | 3886.2 | 6 | 1.544 | 50.7 |
| BICESTER | -1.15254 | 51.9016 | 8.6 | 30474 | 3551.3 | 0 | 0.000 | 59.4 |
| BILLINGHAM_STOCKTON-ON-TEES | -1.29645 | 54.58787 | 34.8 | 110361 | 3173.1 | 58 | 18.279 | 48.6 |
| BIRMINGHAM_WOLVERHAMPTON_DUDLEY^2^ | -2.00965 | 52.53300 | 604.8 | 2266297 | 3747.1 | 599 | 159.858 | 35.6 |
| BISHOP'S STORTFORD | 0.15834 | 51.87227 | 8.9 | 33491 | 3749.6 | 6 | 1.600 | 59.5 |
| BLACKBURN | -2.48227 | 53.74772 | 33.2 | 122993 | 3700.0 | 4 | 1.081 | 51.9 |
| BLACKWOOD | -3.20076 | 51.66582 | 19.2 | 43556 | 2265.6 | 2 | 0.883 | 59.7 |
| BOURNEMOUTH^1^ | -1.87560 | 50.72284 | 116.9 | 371454 | 3177.5 | 230 | 72.384 | 42.3 |
| BRACKNELL | -0.74404 | 51.41576 | 20.9 | 72416 | 3467.0 | 22 | 6.346 | 45.4 |
| BRAINTREE | 0.55071 | 51.87862 | 10.6 | 37603 | 3546.6 | 1 | 0.282 | 65.1 |
| BRIDGEND/PEN-Y-BONT AR OGWR | -3.57292 | 51.51013 | 16.6 | 46112 | 2781.6 | 1 | 0.360 | 62.3 |
| BRIGHOUSE_ELLAND_HUDDERSFIELD_HALIFAX | -1.82019 | 53.68947 | 82.6 | 243098 | 2942.0 | 43 | 14.616 | 62.0 |
| BRIGHTON_PORTSLADE-BY-SEA_SOUTHWICK_SHOREHAM_BY_SEA | -0.23986 | 50.83234 | 64.9 | 293068 | 4519.0 | 171 | 37.840 | 30.6 |
| BRISTOL^1^ | -2.58073 | 51.45001 | 142.5 | 593623 | 4166.0 | 304 | 72.971 | 38.2 |
| BROMSGROVE | -2.06326 | 52.33416 | 14.6 | 39759 | 2728.4 | 5 | 1.833 | 61.5 |
| BURTON UPON TRENT | -1.64688 | 52.81616 | 20.6 | 60847 | 2945.0 | 12 | 4.068 | 54.5 |
| BURY ST EDMUNDS | 0.71596 | 52.24917 | 13.9 | 35590 | 2562.9 | 0 | 0.000 | 51.2 |
| CAERPHILLY | -3.21609 | 51.57943 | 15.2 | 37367 | 2462.6 | 3 | 1.218 | 68.7 |
| CAMBRIDGE | 0.13840 | 52.20763 | 29.9 | 120015 | 4017.1 | 7 | 1.743 | 49.1 |
| CANNOCK | -2.02777 | 52.6915 | 29.6 | 79621 | 2689.0 | 12 | 4.463 | 52.1 |
| CANVEY ISLAND | 0.59342 | 51.51958 | 9.4 | 36050 | 3848.2 | 18 | 4.677 | 38.4 |
| CARDIFF/CAERDYDD | -3.17585 | 51.48296 | 78.3 | 313098 | 4000.0 | 47 | 11.750 | 42.1 |
| CARLISLE | -2.92800 | 54.89573 | 23.1 | 69645 | 3016.6 | 3 | 0.995 | 51.5 |
| CHELMSFORD | 0.46699 | 51.73228 | 28.2 | 97539 | 3455.1 | 11 | 3.184 | 60.7 |
| CHELTENHAM^1^ | -2.06977 | 51.89735 | 28.6 | 106524 | 3725.5 | 28 | 7.516 | 44.6 |
| CHESTER | -2.89277 | 53.19538 | 22.7 | 76220 | 3357.7 | 4 | 1.191 | 66.0 |
| CHESTERFIELD | -1.42488 | 53.23814 | 36.9 | 90174 | 2442.2 | 35 | 14.331 | 62.8 |
| CHIPPENHAM | -2.11833 | 51.46065 | 10.8 | 33839 | 3126.1 | 1 | 0.320 | 38.8 |
| CHURCH_CLAYTON-LE-MOORS_ACCRINGTON | -2.37496 | 53.75943 | 22.4 | 63202 | 2821.8 | 4 | 1.418 | 63.1 |
| CLACTON-ON-SEA | 1.15845 | 51.78969 | 17.7 | 52553 | 2967.2 | 0 | 0.000 | 43.6 |
| CLEETHORPES_GRIMSBY | -0.04444 | 53.56312 | 35.7 | 124432 | 3485.3 | 56 | 16.067 | 44.3 |
| CORBY | -0.69911 | 52.48392 | 14.6 | 45049 | 3095.2 | 2 | 0.646 | 60.3 |
| COVENTRY_BEDWORTH^1^ | -1.49334 | 52.44251 | 89.4 | 341295 | 3818.2 | 79 | 20.690 | 53.1 |
| CRAWLEY | -0.18493 | 51.11271 | 23.8 | 100808 | 4228.3 | 19 | 4.494 | 60.8 |
| CREWE | -2.43864 | 53.10082 | 17.1 | 60068 | 3504.4 | 7 | 1.998 | 56.3 |
| CWMBRAN | -3.02666 | 51.65082 | 21.5 | 55001 | 2554.8 | 0 | 0.000 | 56.3 |
| DARLINGTON | -1.55045 | 54.52905 | 24.0 | 82822 | 3455.0 | 54 | 15.629 | 46.0 |
| DERBY | -1.48220 | 52.91337 | 63.7 | 242196 | 3801.7 | 90 | 23.673 | 46.5 |
| DONCASTER_BENTLEY | -1.12655 | 53.53111 | 41.7 | 105684 | 2536.4 | 3 | 1.183 | 59.2 |
| EASTBOURNE | 0.28293 | 50.76561 | 25.8 | 95999 | 3726.4 | 48 | 12.881 | 52.4 |
| EASTLEIGH | -1.36644 | 50.97094 | 27.4 | 71001 | 2592.2 | 7 | 2.700 | 60.7 |
| ELLESMERE PORT | -2.92084 | 53.28739 | 17.9 | 57488 | 3206.7 | 34 | 10.603 | 40.8 |
| ESTON_MIDDLESBROUGH | -1.18378 | 54.56247 | 49.7 | 168429 | 3391.5 | 17 | 5.013 | 51.1 |
| EXETER | -3.52457 | 50.71933 | 32.6 | 108922 | 3339.3 | 33 | 9.882 | 51.8 |
| FLEET | -0.84677 | 51.27715 | 11.1 | 32914 | 2977.6 | 6 | 2.015 | 46.9 |
| FOLKESTONE | 1.17990 | 51.08067 | 14.4 | 45163 | 3136.8 | 12 | 3.826 | 31.7 |
| FRIMLEY_CAMBERLEY_FARNHAM | -0.74979 | 51.29149 | 77.9 | 224005 | 2876.9 | 70 | 24.331 | 46.1 |
| GILLINGHAM_CHATHAM | 0.54952 | 51.37085 | 44.1 | 191831 | 4352.2 | 77 | 17.692 | 47.0 |
| GLOUCESTER^1^ | -2.23969 | 51.86717 | 31.9 | 114907 | 3602.9 | 14 | 3.886 | 47.7 |
| GRANTHAM | -0.64107 | 52.91315 | 13.5 | 36950 | 2730.8 | 0 | 0.000 | 53.5 |
| GRAYS | 0.32696 | 51.47861 | 16.5 | 67300 | 4083.4 | 12 | 2.939 | 57.7 |
| GREAT YARMOUTH | 1.72836 | 52.60644 | 11.8 | 37235 | 3165.6 | 6 | 1.895 | 47.3 |
| HARROGATE | -1.52741 | 53.99072 | 26.6 | 77266 | 2906.7 | 5 | 1.720 | 59.8 |
| HARTLEPOOL | -1.20992 | 54.68486 | 26.0 | 82334 | 3163.0 | 6 | 1.897 | 32.0 |
| HEMEL HEMPSTEAD | -0.47111 | 51.74553 | 20.9 | 78276 | 3750.2 | 34 | 9.066 | 54.6 |
| HIGH WYCOMBE_BEACONSFIELD | -0.69196 | 51.61583 | 43.1 | 105164 | 2438.9 | 18 | 7.380 | 66.3 |
| HORNDEAN_HAVANT | -0.99079 | 50.88398 | 45.5 | 149597 | 3286.9 | 35 | 10.648 | 53.8 |
| HORSHAM | -0.32356 | 51.06343 | 14.6 | 46537 | 3186.8 | 12 | 3.765 | 59.7 |
| HOUGHTON-LE-SPRING | -1.47308 | 54.84076 | 16.0 | 38207 | 2382.1 | 0 | 0.000 | 74.2 |
| IPSWICH | 1.15929 | 52.05868 | 49.1 | 153272 | 3124.8 | 9 | 2.880 | 47.9 |
| KETTERING | -0.72379 | 52.40064 | 19.6 | 56465 | 2884.6 | 0 | 0.000 | 51.7 |
| KIDDERMINSTER | -2.24086 | 52.38932 | 19.0 | 54974 | 2892.5 | 8 | 2.766 | 47.8 |
| KING'S LYNN | 0.40263 | 52.75615 | 10.6 | 32408 | 3062.7 | 0 | 0.000 | 57.1 |
| KINGSTON UPON HULL | -0.34280 | 53.73984 | 29.9 | 121534 | 4070.6 | 130 | 31.936 | 37.9 |
| LANCASTER | -2.79632 | 54.04242 | 26.1 | 95465 | 3654.4 | 5 | 1.368 | 44.3 |
| LEEDS_WAKEFIELD_BRADFORD | -1.59534 | 53.75756 | 360.2 | 1239006 | 3439.5 | 356 | 103.503 | 65.2 |
| LEICESTER^1^ | -1.13063 | 52.63678 | 127.3 | 450433 | 3537.6 | 109 | 30.812 | 51.5 |
| LEIGHTON BUZZARD | -0.65463 | 51.9172 | 9.5 | 30246 | 3197.0 | 0 | 0.000 | 55.5 |
| LETCHWORTH GARDEN CITY | -0.21392 | 51.97383 | 13.2 | 37291 | 2815.6 | 2 | 0.710 | 54.0 |
| LEYLAND | -2.69120 | 53.68944 | 36.8 | 91405 | 2482.0 | 1 | 0.403 | 61.9 |
| LIVERPOOL | -2.93735 | 53.40423 | 219.3 | 831194 | 3791.1 | 287 | 75.705 | 48.0 |
| LLANELLI | -4.14724 | 51.68477 | 12.2 | 31467 | 2580.4 | 6 | 2.325 | 45.1 |
| LONDON | -0.09668 | 51.51410 | 1533.1 | 8481656 | 5532.3 | 3890 | 703.142 | 30.6 |
| LOUGHBOROUGH | -1.20540 | 52.77243 | 17.0 | 55317 | 3263.8 | 8 | 2.451 | 60.0 |
| LOWESTOFT | 1.75234 | 52.47380 | 12.5 | 36319 | 2912.9 | 28 | 9.612 | 51.6 |
| LUTON_DUNSTABLE_HOUGHTON REGIS | -0.48001 | 51.88928 | 53.3 | 244749 | 4590.9 | 69 | 15.030 | 43.2 |
| MACCLESFIELD | -2.13583 | 53.25905 | 22.4 | 55156 | 2460.6 | 0 | 0.000 | 54.5 |
| MAIDENHEAD | -0.72468 | 51.52248 | 16.3 | 49999 | 3063.0 | 6 | 1.959 | 55.7 |
| MAIDSTONE | 0.52165 | 51.27139 | 28.0 | 90729 | 3244.6 | 21 | 6.472 | 55.1 |
| MANCHESTER_SALFORD | -2.28285 | 53.47781 | 703.3 | 2399014 | 3411.0 | 627 | 183.814 | 62.3 |
| NELSON_BURNLEY_BRIERFIELD | -2.22648 | 53.81537 | 36.9 | 121158 | 3287.8 | 2 | 0.608 | 56.9 |
| NEWCASTLE UPON TYNE | -1.63026 | 54.98723 | 126.1 | 477302 | 3784.9 | 190 | 50.199 | 41.1 |
| NEWPORT PAGNELL | -0.72708 | 52.08553 | 75.3 | 199640 | 2650.6 | 28 | 10.564 | 53.6 |
| NEWPORT/CASNEWYDD | -2.98765 | 51.58556 | 24.0 | 65263 | 2724.7 | 11 | 4.037 | 56.9 |
| NORTHAMPTON | -0.90167 | 52.23322 | 62.1 | 204444 | 3290.8 | 19 | 5.774 | 46.1 |
| NORTHFLEET_GRAVESEND | 0.35376 | 51.44130 | 19.6 | 77192 | 3931.9 | 21 | 5.341 | 49.1 |
| NORTHWICH | -2.51230 | 53.26072 | 16.2 | 36643 | 2262.4 | 7 | 3.094 | 62.3 |
| NORWICH | 1.31029 | 52.62706 | 54.3 | 188607 | 3473.6 | 127 | 36.562 | 50.5 |
| NOTTINGHAM^1^ | -1.15005 | 52.95033 | 148.2 | 571241 | 3853.5 | 138 | 35.812 | 51.5 |
| NUNEATON | -1.46329 | 52.52375 | 23.4 | 71457 | 3052.9 | 6 | 1.965 | 55.3 |
| OXFORD | -1.24407 | 51.75173 | 39.5 | 151827 | 3844.3 | 31 | 8.064 | 50.8 |
| PAIGNTON | -3.56110 | 50.43561 | 31.1 | 106467 | 3427.0 | 20 | 5.836 | 43.8 |
| PETERBOROUGH | -0.25422 | 52.59014 | 35.7 | 115260 | 3229.2 | 48 | 14.864 | 51.8 |
| PLYMOUTH | -4.14039 | 50.37440 | 58.8 | 229123 | 3898.0 | 5 | 1.283 | 38.1 |
| PORTSMOUTH | -1.09332 | 50.79491 | 25.0 | 166062 | 6647.9 | 88 | 13.237 | 51.3 |
| PRESTON | -2.68061 | 53.75564 | 34.2 | 119579 | 3496.1 | 14 | 4.004 | 61.0 |
| RAMSGATE | 1.42250 | 51.33650 | 32.8 | 115746 | 3526.5 | 49 | 13.895 | 43.5 |
| RAYLEIGH | 0.60625 | 51.58464 | 67.8 | 255856 | 3775.8 | 88 | 23.306 | 54.6 |
| REDDITCH | -1.92827 | 52.30925 | 26.9 | 71513 | 2654.9 | 18 | 6.780 | 59.6 |
| REIGATE_REDHILL | -0.18764 | 51.23957 | 17.9 | 52923 | 2962.3 | 33 | 11.140 | 65.8 |
| RHYL_PRESTATYN | -3.45526 | 53.32516 | 14.0 | 41709 | 2972.3 | 10 | 3.364 | 56.0 |
| RUGBY | -1.26489 | 52.37141 | 19.2 | 52490 | 2739.6 | 4 | 1.460 | 55.1 |
| RUNCORN | -2.73984 | 53.34049 | 16.2 | 50962 | 3148.0 | 0 | 0.000 | 41.7 |
| SALISBURY | -1.78416 | 51.07363 | 15.8 | 39738 | 2517.1 | 0 | 0.000 | 55.9 |
| SCARBOROUGH | -0.40381 | 54.28023 | 13.6 | 44517 | 3268.4 | 4 | 1.224 | 45.0 |
| SCUNTHORPE | -0.65171 | 53.58288 | 20.3 | 64189 | 3164.4 | 6 | 1.896 | 55.9 |
| SHEFFIELD | -1.40731 | 53.40658 | 202.2 | 663329 | 3280.7 | 149 | 45.417 | 46.6 |
| SHREWSBURY | -2.73506 | 52.70204 | 25.2 | 60761 | 2412.1 | 1 | 0.415 | 60.2 |
| SITTINGBOURNE | 0.74129 | 51.33972 | 14.0 | 39819 | 2847.6 | 16 | 5.619 | 54.8 |
| SKELMERSDALE | -2.77378 | 53.55903 | 10.9 | 31536 | 2906.0 | 5 | 1.721 | 55.8 |
| SOUTHAMPTON | -1.40287 | 50.90547 | 54.0 | 254118 | 4705.0 | 39 | 8.289 | 59.1 |
| ST ALBANS | -0.33548 | 51.75421 | 30.2 | 82426 | 2730.8 | 15 | 5.493 | 53.6 |
| STEVENAGE | -0.20784 | 51.90369 | 25.9 | 83336 | 3214.5 | 2 | 0.622 | 50.8 |
| STOKE-ON-TRENT | -2.18051 | 53.00306 | 105.1 | 307707 | 2927.6 | 74 | 25.277 | 45.3 |
| SUNDERLAND | -1.38761 | 54.90499 | 34.6 | 118893 | 3438.3 | 7 | 2.036 | 45.3 |
| SUTTON IN ASHFIELD_MANSFIELD | -1.21168 | 53.14564 | 50.4 | 131005 | 2601.6 | 19 | 7.303 | 56.9 |
| SWADLINCOTE | -1.55157 | 52.77173 | 12.3 | 34807 | 2832.1 | 4 | 1.412 | 55.4 |
| SWINDON | -1.76405 | 51.56046 | 49.9 | 173888 | 3482.7 | 93 | 26.704 | 48.1 |
| TAMWORTH | -1.69227 | 52.64226 | 21.4 | 68747 | 3210.1 | 3 | 0.935 | 50.9 |
| TAUNTON | -3.10290 | 51.01881 | 19.3 | 57286 | 2973.3 | 5 | 1.682 | 48.4 |
| TELFORD | -2.46540 | 52.69416 | 50.7 | 121934 | 2405.6 | 5 | 2.078 | 57.6 |
| THATCHAM_NEWBURY | -1.29261 | 51.40157 | 17.4 | 53744 | 3082.0 | 5 | 1.622 | 57.3 |
| THORNTON_POULTON-LE-FLYDE-CLEVELEYX-FLEETWOOD | -3.01169 | 53.87751 | 73.9 | 250077 | 3385.8 | 27 | 7.975 | 46.2 |
| TROWBRIDGE | -2.20056 | 51.32365 | 13.2 | 37332 | 2837.2 | 3 | 1.057 | 51.6 |
| WARRINGTON | -2.59075 | 53.39359 | 49.0 | 146227 | 2985.7 | 16 | 5.359 | 51.6 |
| WELLINGBOROUGH | -0.69094 | 52.30247 | 12.5 | 43245 | 3454.9 | 1 | 0.289 | 59.1 |
| WELWYN GARDEN CITY_HATFIELD | -0.21319 | 51.77777 | 29.0 | 85211 | 2937.2 | 12 | 4.086 | 51.1 |
| WHITNASH_ROYAL LEMINGTON SPA_WARWICK | -1.54891 | 52.27936 | 20.1 | 77349 | 3845.6 | 22 | 5.721 | 59.3 |
| WINCHESTER | -1.31482 | 51.06157 | 14.2 | 40379 | 2852.7 | 0 | 0.000 | 76.0 |
| WOKINGHAM_READING | -0.90302 | 51.43343 | 86.4 | 286048 | 3310.6 | 92 | 27.789 | 60.1 |
| WORCESTER | -2.22092 | 52.19236 | 31.6 | 94609 | 2990.2 | 26 | 8.695 | 48.4 |
| WORKSOP | -1.12298 | 53.30945 | 11.6 | 34633 | 2976.0 | 2 | 0.672 | 66.2 |
| WORTHING | -0.37058 | 50.81325 | 30.9 | 127821 | 4131.3 | 104 | 25.174 | 42.7 |
| WREXHAM/WRECSAM | -2.99689 | 53.04800 | 24.1 | 60361 | 2508.2 | 7 | 2.791 | 57.8 |
| YEOVIL | -2.63232 | 50.94271 | 14.2 | 41192 | 2893.1 | 17 | 5.876 | 54.3 |
| YORK | -1.07985 | 53.96450 | 35.8 | 139098 | 3885.0 | 20 | 5.148 | 56.2 |

^1^ Cities where fox density (family groups km^-2^) was measured by Harris & Smith (1987) and which are comparable to conurbations identified in the present study

^2^ Cities where fox density (family groups km^-2^) was measured by Harris & Smith (1987) but which have merged since the 1980s and which are therefore not comparable to conurbations identified in the present study
