# Supplementary material for: Changes in the Distribution of Red Foxes (Vulpes vulpes) in Urban Areas in Great Britain: Findings and Limitations of a Media-Driven Nationwide Survey
Source: PLoS One. 2014 Jun 11;9(6):e99059. doi: 10.1371/journal.pone.0099059 (PMC4053368; doi:10.1371/journal.pone.0099059)
Supplement: Licence S1 — Digimap licence agreement. (DOCX) [file pone.0099059.s002.docx]

Digimap licence agreement can be found at : <http://digimap.edina.ac.uk/webhelp/os/copyright/licence_agreement.htm#sublicence>

As stated on all the figures/maps.

Copy of this is below:

| **From 1 August 2009 this Sub-Licence Agreement is subject to a Variation Agreement, which alters some of the terms in the original Sub-Licence Agreement. Both Sub-Licence and Variation Sub-Licence must be read in conjunction with one another and both are given here. Use the following links to jump to each one:**   - [**Second Variation Agreement**](http://digimap.edina.ac.uk/webhelp/os/copyright/licence_agreement.htm#secondvariation) - [**Variation Agreement**](http://digimap.edina.ac.uk/webhelp/os/copyright/licence_agreement.htm#variation) - [**Sub-Licence Agreement**](http://digimap.edina.ac.uk/webhelp/os/copyright/licence_agreement.htm#sublicence)  Second Variation Agreement to the Ordnance Survey Data Sub-Licence Agreement Effective from 1 August 2010 - 31 July 2011  **VARIATION AGREEMENT to THE ORDNANCE SURVEY DATA SUB-LICENCE AGREEMENT between the JISC Content Procurement Company Limited and you, the Sub Licensee.**  **THIS VARIATION AGREEMENT is made as of [insert date], the (Effective Date).**  **Between:**   1. **THE JISC CONTENT PROCUREMENT COMPANY LIMITED TRADING AS JISC COLLECTIONS**(company registration number 05747339) a company incorporated in England and Wales and limited by guarantee whose registered office is at Ground Floor, Brettenham House South, Lancaster Place, LONDON, WC2E 7EN (JISC Collections); and 2. ?INSTITUTION NAME? (Sub Licensee)   **RECITALS:**  **WHEREAS** by a Data Sub-Licence Agreement between JISC Collections and you as ‘Sub Licensee’ (the ‘Existing Agreement’), JISC Collections granted you access to and use of Ordnance Survey Data in accordance with the terms of the Existing Agreement;  **WHEREAS** the parties agreed to vary the Existing Agreement as set forth in the first variation agreement (‘Variation Agreement’);  **WHEREAS** the parties have now agreed to vary the Existing Agreement as set forth in this agreement (‘Second Variation Agreement’).  **IT IS NOW AGREED AS FOLLOWS:** AGREEMENT **1.1** In consideration of the fee as referred to below, the parties hereto agree to amend the Existing Agreement as of 14th July 2010, as set forth below. 2 THE AMENDMENTS **2.1** The term shall be extended to the 31st July 2011.  **2.2** The following shall be inserted at the end of Clause 9.2 as a new sub-paragraph (b):  "This Agreement shall automatically terminate on the coming into force of a new Agreement between Ordnance Survey and JISC Collections expressly replacing this Agreement.  **2.3** OS VectorMap Local shall be included in the Licensed Work and shall accordingly be included in the list of products at Appendix 2. 3. GENERAL **3.1** Except as specifically amended hereby, all of the existing terms and conditions of the Existing Agreement, as amended, are hereby ratified and remain in effect without revision.  **3.2** To the extent any terms or conditions of the Existing Agreement, as amended, conflict with or are inconsistent with this Second Variation Agreement, the terms of this Second Variation Agreement shall prevail.  **3.3** The parties agree that the Existing Agreement, the Variation Agreement and this Second Variation Agreement and any documents referred to in it constitute the entire agreement and understanding between the parties concerning the subject matter of the Existing Agreement. The Existing Agreement, as amended, and this Second Variation Agreement supersede all understandings, representations and agreements made between the parties concerning such subject matter. However neither party seeks to exclude liability for any fraudulent misrepresentations.  **3.4** This Second Variation Agreement is governed by and construed in accordance with English law. The parties further agree that the Second Variation Agreement will be subject to, and within the jurisdiction of, the English Courts.   Variation Agreement to the Ordnance Survey Data Sub-Licence Agreement **Effective from 1 August 2009 - 31 July 2010**  **VARIATION AGREEMENT to THE ORDNANCE SURVEY DATA SUB-LICENCE AGREEMENT** between the JISC Content Procurement Company Limited and you, the Sub Licensee.  **THIS VARIATION AGREEMENT** is made as of 1st August 2009, the (**Effective Date**). **This Variation Agreement is made**  **Between:**   1. **THE JISC CONTENT PROCUREMENT COMPANY LIMITED (TRADING AS JISC COLLECTIONS**(company registration number 05747339) a company incorporated in England and Wales and limited by guarantee whose registered office is at Ground Floor, Brettenham House South, Lancaster Place, LONDON, WC2E 7EN (JISC Collections); 2. 'INSTITUTION NAME' (Sub Licensee)   **RECITALS:**  **WHEREAS** by a Data Sub-Licence Agreement between JISC Collections and you as ‘Sub Licensee’ dated 1st August 2009 (the ‘Existing Agreement’), JISC Collections granted you access to and use of Ordnance Survey Data in accordance with the terms of the Existing Agreement;  **WHEREAS** the parties have now agreed to vary the Existing Agreement as set forth in this agreement (‘Variation Agreement’).  **IT IS NOW AGREED AS FOLLOWS:** 1 AGREEMENT **1.1** In consideration of the fee as referred to below, the parties hereto agree to amend the Existing Agreement as of the 1st August 2009, as set forth below. 2 THE AMENDMENTS **2.1** Save for the amended definition of ‘Authorised Users’ as set out in paragraph 2.2 below, all capitalised and defined terms used in this Variation Agreement shall have the meanings ascribed to them in the Existing Agreement.  **2.2** The definition of ‘Authorised Users’ in the Existing Agreement, is hereby deleted and replaced by the following: ‘means any students of, staff (both current or retired) of, or visitors to an Authorised Institution who are both properly:   1. authorised by an Authorised Institution to access the Authorised Institution’s electronic information services via secure authentication; **and** 2. registered with a Datacentre in accordance with the procedure as set out in Schedule 7 of the Existing Agreement;   for the avoidance of doubt, students who are registered at an overseas campus of an Authorised Institution (**‘Overseas Students’**) **do not** qualify as ‘Authorised Users’ of that Authorised Institution whilst they are overseas (studying or otherwise) for the purposes of this Variation Agreement. However, Overseas Students may qualify as ‘Authorised Users’ during any period spent studying in the UK at an Authorised Institution. Overseas Students will lose their Authorised User status as soon as they leave the United Kingdom.  **2.3** Any reference to Athens in the Existing Agreement is hereby amended to read ‘UK Access Management Federation’.  **2.4** The parties have agreed to delete the following of paragraph 4 of Appendix 3 of the Existing Agreement, the User Registration section: ‘Where a class registration is needed, require each member of the class to sign a class registration form, and to identify the Athens accounts to be used for class registration. Note that class registration is not possible where an institution implements authentication and authorisation systems involving Athens Devolved Authentication or UK Access Management Federation. Require the class lecturer to be the Responsible Officer for use of the Licensed Work and Digital Maps under class registration. Identify which Athens account is to be allocated to the class lecturer nominated as the Responsible Officer for class registration. Require each class registration to be terminated after three (3) Months’.  **2.5** An additional Clause 3.1.16 shall be added and shall be as follows:  "3.1.16share data with other Authorised Users of both the same Authorised Institution and different Authorised Institutions"  **2.6** The term as set out in Clause 9.1 of the Existing Agreement shall hereby be extended until 31 July 2010.  **2.7** Schedule 1 to this Variation Agreement shall supersede and replace Appendix 2 (Licensed Work) of the Existing Agreement, which such Appendix 2 to the Existing Agreement shall be without effect.  **2.8** Schedule 2 to this Variation Agreement shall supersede and replace Appendix 4 of the Existing Agreement, which such Appendix 4 to the Existing Agreement shall be without effect.  **2.9** Any reference in the Existing Agreement to Ordnance Survey’s discontinued Land Line ® data product shall be deleted. The parties hereby agree that any continued use of Land-Line will be subject to the relevant party entering into an archive licence or residual rights licence for Land-Line with Ordnance Survey. 3 GENERAL **3.1** Except as specifically amended hereby, all of the existing terms and conditions of the Existing Agreement, as amended, are hereby ratified and remain in effect without revision.  **3.2** To the extent any terms or conditions of the Existing Agreement, as amended, conflict with or are inconsistent with this Variation Agreement, the terms of this Variation Agreement shall prevail.  **3.3** The parties agree that the Existing Agreement and this Variation Agreement and any documents referred to in it constitute the entire agreement and understanding between the parties concerning the subject matter of the Existing Agreement. The Existing Agreement, as amended, and this Variation Agreement supersede all understandings, representations and agreements made between the parties concerning such subject matter. However neither party seeks to exclude liability for any fraudulent misrepresentations.  **3.4** This Variation Agreement is governed by and construed in accordance with English law. The parties further agree that the Variation Agreement will be subject to, and within the jurisdiction of, the English Courts.  AS WITNESS the hands of the parties the day and year first above written.   \| Having read and understood this Variation Agreement, signed for and on behalf of **The JISC Content Procurement Company Limited (trading as JISC Collections)** \| \| Having read and understood this Agreement signed for and on behalf of [**the Sub Licensee**] \| \| \| --- \| --- \| --- \| --- \| \| **Signature** \| ............................................... \| **Signature** \| ............................................... \| \| **Name** \| ............................................... \| **Name** \| ............................................... \| \| **Title** \| ............................................... \| **Title** \| ............................................... \| \| **Date** \| ............................................... \| **Date** \| ............................................... \|    Schedule 1 of the Variation Agreement (amending Appendix 2 of the Existing Agreement) APPENDIX 2 - LICENSED WORK  The Licensed Work consists of the following products:   - OS MasterMap® Topography Layer - OS MasterMap® Integrated Transport Network Layer - 1:10 000 Scale Colour Raster - 1:25 000 Scale Colour Raster - 1:50 000 Scale Colour Raster - 1:50 000 Scale Gazetteer - 1:250 000 Scale Colour Raster - Meridian 2 - Strategi® - MiniScale® - Code-Point® - Code-Point® with polygons - Land-Form PROFILE® contours and Digital Terrain Mapping - Land-Form PANORAMA contours and Digital Terrain Mapping - Boundary-Line    Schedule 2 of the Variation Agreement (amending Appendix 4 of the Existing Agreement) APPENDIX 4 - **Paper and Electronic Publication Sizes**  **Paper and Electronic Publication Sizes**  All Digital Maps, whether in paper or electronic format, employed for either Academic or Institutional Administrative Use, must carry the following copyright notice:  © Crown Copyright/database right 20(yy). An Ordnance Survey/EDINA supplied service. Academic usePaper Publication  \| **Publication type** \| **Dataset** \| **Maximum Size per image** \| **Maximun Ordnance Survey Mapping (% of total publication content)** \| \| --- \| --- \| --- \| --- \| \| **- Conference papers - Academic articles - Journals - Dissertations and theses - Booklets** \| All Dataset \| 1 250cm^2^ (A3) \| 45% \| \| **- Course notes** \| All Dataset \| No Size limitations \| 45% \| \| **- Academic Posters** \| All Dataset \| No Size limitations \| 100% \| |
| --- | --- | --- | --- | --- | --- | --- | --- | --- | --- | --- | --- | --- | --- | --- | --- | --- | --- | --- | --- | --- | --- | --- | --- | --- | --- | --- | --- | --- | --- | --- | --- | --- | --- | --- | --- | --- |

## Electronic Publication

Electronic Publication refers to publication on DVD, CD_ROM, memory stick or any other form of storage media including the internet.

This mode of publication is an electronic rendition of the paper form, solely for ease of delivery or dissemination. Digital Maps must therefore only appear as static images and must not be created in real time for serving to the reader.

Paper publication size limits are applicable in all cases.

## Repositories

Repositories (centralised storage systems) may be operated by Authorised Institutions to hold and make available Electronic Publications.

It is understood that some repositories may make such publications available to members of the public as well as to members of the Authorised Institution.

Digital Maps (but not Digital Data) may be included in documents held in repositories as long as those documents are in a locked format, for example, pdf. Documents must be locked in such a way that the Digital Maps cannot be extracted for reuse outside the terms of the Ordnance Survey Data Sub-Licence Agreement.

Paper publication size limits are applicable in all cases.

For the avoidance of doubt, Digital Data must never be stored in and made available from any repository operated centrally by or for an institution or subject community.

### lntranet Publication

| **Publication type** | **Dataset type** | **Maximum mapped area per image** |
| --- | --- | --- |
| - Conference papers - Academic articles - Journals - Course notes - Dissertations and theses - Booklets | All datasets | No size limitations |

An Intranet is an internal network specific to a single Higher and Further Education Institution and only accessible to members of that Institution. This network is **NOT** connected to the public Internet. For **Internet** publication see table below.

### Internet Publication

| **Publication type** | **Dataset type** | **Maximum mapped area per image** |
| --- | --- | --- |
| - Conference papers - Academic articles - Journals - Course notes - Dissertations and theses - Booklets - Digital Maps on a web page, project website or similar | All datasets | 1 048 576 pixels |

When rendering mapping on a website:

- Only Digital Maps may be published. Digital Data and mapping in GeoPDF format may not be published at any time.
- It must only be available as an image and not be accompanied by drawing or measuring tools.
- It is permissible to zoom in and out to enlarge or reduce the viewing scale of a discrete map image but not to change from one dataset to another of higher/lower resolution.
- It is permissible to pan to the edge of a discrete map image (where the ‘viewing frame’ is smaller than the overall image).
- Digital Maps may be displayed at any size on screen.
- More than one Digital Map may be included but no single Digital Map may be of a size greater than specified above.

### Institutional Administrative Use

These criteria apply to paper, electronic and internet publication.

| Paper brochures and leaflets | All datasets | Single image up to 1250 cm^2^ (A3) |
| --- | --- | --- |
| Paper display and promotion, (display boards, posters) | All datasets | No size limitations |
| Electronic publication | All datasets | See instructions below |
| Intranet | All datasets | No size limitations |
| Internet | All datasets | No size limitations |

Institutional Administrative Use shall only include:

- Showing the extent and/or locations of the Institution’s campus
- Providing directional guidance or a route showing how to locate any premises owned or leased by the institution. This can be:
- General directional information applicable at all times, for example, directions to a specific department or to a specific office of the institution.
- Directions linked to specific events organised by the institution itself on the institution’s premises.

Institutional Administrative Use are subject to the following rules:

- Only the amount of mapping necessary to meet the specific need should be used
- The mapping must include overlay details relating to the purpose for which it is being used by the academic institution.

### Electronic Publication

Electronic Publication refers to publication on DVD, CD_ROM, memory stick or any other form of storage media.

This mode of publication is an electronic rendition of the paper form, solely for ease of delivery or dissemination. Digital Maps must therefore only appear as static images and must not be created in real time for serving to the reader.

Paper publication size limits are applicable in all cases.

### Ordnance Survey Data Sub-Licence Agreement

THE JISC CONTENT PROCUREMENT COMPANY LIMITED (TRADING AS JISC COLLECTIONS)(Company Number 05747339), a company incorporated in England and Wales and limited by guarantee whose registered offices is at Ground Floor, Brettenham House South, Lancaster Place, London WC2E 7EN

OFFERS to you, the Sub-Licensee, the permission to access and use the Licensed Work and Digital Maps on the terms and conditions as set out in this Sub-Licence. Upon completing and returning the enclosed Acceptance of Sub-Licence Form, your institution will become a non-exclusive Sub-Licensee of JISC Collections.

Acceptance shall be by delivery of a completed copy of the Acceptance of Sub-Licence Form attached hereto to JISC Collections or a JISC Collections' authorised agent who will accept delivery on behalf of JISC Collections. Acceptance shall be acceptance of all terms of this Sub-Licence. In the event that no or partial compliance is made as to the manner or form described for acceptance, no sub-licence will be granted and this offer is deemed withdrawn.

## RECITALS

**WHEREAS** the Ordnance Survey Data comprises the following products: OS MasterMap® - Topographic Layer, OS MasterMap® - Integrated Transport Network Layer, Land-Line.Plus®, 1:10 000 Scale Colour Raster, 1:25 000 Scale Colour Raster, 1:50 000 Scale Colour Raster, 1:50 000 Scale Gazetteer, Strategi®, Meridian 2™, Code-Point®, Code-Point® with polygons, Land-Form PROFILE® - contours & Digital Terrain Mapping, Land-Form PANORAMA® - contours & Digital Terrain Mapping and Boundary-Line™ ("Licensed Work");

**AND WHEREAS** the Ordnance Survey Data and all Intellectual Property Rights (as hereafter defined) therein are owned by or licensed to the Secretary of State (acting through Ordnance Survey) ("Ordnance Survey");

**AND WHEREAS** by an agreement between Ordnance Survey, HEFCE (as hereafter defined) and JISC Collections (the "Ordnance Survey Data Supply and Licence Agreement") dated 12 July 2007, JISC Collections is permitted to sub-license the access and use of Ordnance Survey Data to the Sub-Licensees in accordance with the terms of this Sub-Licence;

JISC COLLECTIONS AND THE SUB-LICENSEE AGREE AS FOLLOWS:

## 1. DEFINITIONS

1.1 In this Sub-Licence, the following expressions shall have the following meanings:

"Authorised Users"

means individuals who are registered with the Sub-Licensee and the appointed Datacentre from time to time in accordance with the procedure as set out in Appendix 3 and have been authorised by the Sub-Licensee to access the Sub-Licensee's electronic information services whether on-site or off-site via Secure Authentication and who are affiliated to the Sub-Licensee as a current student, faculty member or employee of the Sub-Licensee. Persons who are not a current student, faculty member or an employee of the Sub-Licensee, but who are permitted to access the Sub-Licensee's electronic information services whilst involved in teaching, education or research at an Institution or who are a current student of an Institution ("Walk-In Users") are also deemed to be Authorised Users, only for the time they are within the Library Premises.

"Crown Copyright Fee"

means the fee payable to Ordnance Survey by the Sub-Licensee for use of digital data which are still in copyright to the Crown as set out in Appendix 1.

"Commercial Use"

means the use of the whole or parts of the Licensed Work and Digital Maps for any reason which generates a profit.

"Datacentre"

means a datacentre appointed from time to time by HEFCE. At the time of this agreement the appointed datacentre is EDINA which is the national datacentre based at Edinburgh University Data

"Digital Data"

means that part of the Licensed Work that will be made available via Secure Authentication as digital data and not as Digital Maps.

"Digital Maps"

means any or all of the maps created by a Datacentre from the Licensed Work to be used in a Service provided by a Datacentre.

"Educational Purposes"

means for the purpose of education, teaching, distance learning, private study and/or research.

"Fee"

means the fee as set out in Appendix 1.

"HEFCE"

means the Higher Education Funding Council for England.

"Institution"

means a higher, further education institution or other organisations in the UK whose name appears on the list of eligible institutions as found on <http://www.jisc-collections.ac.uk/jisc_banding.aspx> as the same may be updated from time with the agreement in writing of the Ordnance Survey.

"Intellectual Property Rights"

means patents, trademarks, trade names, design rights, copyright (including rights in computer software and moral rights), database rights, rights in know-how and other intellectual property rights, in each case whether registered or unregistered and including applications for the grant of any of the foregoing and all rights or forms of protection having equivalent or similar effect to any of the foregoing which may subsist anywhere in the world.

"Library Premises"

means the physical premises of the library or libraries operated by the Sub-Licensee.

"Licensed Work"

means the collection of Ordnance Survey's digital data products known to the parties as "Ordnance Survey Data" comprising the products as listed in Appendix 2 and such other Ordnance Survey data products as JISC Collections and Ordnance Survey may agree from time to time.

"Service"

means a service provided by JISC Collections or any Datacentre acting on its behalf such as but not limited to Digimap.

"Secure Authentication"

means access by Athens authentication or Shibboleth technology based authentication or by another means of authentication agreed between Ordnance Survey and JISC Collections from time to time.

"Secure Network"

means a network which is only accessible by Secure Authentication.

"Sub-Licensee"

means the sub-licensee whose details are set out in the Acceptance of Sub-Licence Form attached hereto and made a part hereof.

"Use"

means viewing, copying, adaptation, reproduction, manipulation or modification of the Digital Data to create customised maps and models and any further use of such customised maps and models by Authorised Users in accordance with Clauses 3 and 4 of this Sub-Licence Agreement.

1.2 Headings contained in this Sub-Licence are for reference purposes only and shall not be deemed to be an indication of the meaning of the clause to which they relate.

1.3 Where the context so implies, words importing the singular number shall include the plural and vice versa and words importing the masculine shall include the feminine and vice versa.

## 2. GRANT OF SUB-LICENCE

2.1 The Sub-Licensee agrees to pay the Fee set out in Appendix 1, in consideration of which HEFCE agrees to grant to the Sub-Licensee a non-exclusive revocable sub-licence to access and use the Licensed Work and Digital Maps and to allow Authorised Users to access and use the Licensed Work and Digital Maps throughout the term of this Sub-Licence by Secure Authentication for Educational Purposes only.

2.2 This Sub-Licence supersedes and replaces all previous agreements and licences between the Sub-Licensee and the Higher Education Funding Council for England in relation to the Licensed Work, together with any variations thereto, in their entirety.

## 3. USE OF THE LICENSED WORK(S)

3.1 Subject to the provisions of Appendices 3 and 4, throughout the term of this Sub-Licence the Sub-Licensee may for Educational Purposes only:

3.1.1 make such temporary local electronic copies of parts of the Licensed Work and Digital Maps as are necessary to ensure efficient use by Authorised Users, provided that such use is subject to all the terms and conditions of this Sub-Licence;

3.1.2 allow Authorised Users to access the Licensed Work and Digital Maps by Secure Authentication in order to search, retrieve, display and view, and otherwise use parts thereof including but not limited to Use the Digital Data in accordance with the terms of this Sub-Licence;

3.1.3 allow Authorised Users to electronically save parts of the Licensed Work and Digital Maps;

3.1.4 allow Authorised Users to print out copies of parts of the Licensed Work and Digital Maps;

3.1.5 allow Authorised Users to incorporate parts of the Licensed Work and Digital Maps in printed and electronic course packs, study packs and course notes hosted on a Secure Network, virtual learning environments, managed learning environments and multi-media works. Each item shall carry the following copyright notice "© Crown Copyright/database right 20(yy). An Ordnance Survey/(Datacentre) supplied service." Course packs in non-electronic non-print perceptible form, such as Braille, may also be offered to Authorised Users;

3.1.6 allow Authorised Users to incorporate parts of the Licensed Work and Digital Maps in printed or electronic form in assignments and portfolios, theses and in dissertations ("the Academic Works"), including reproductions of the Academic Works for personal use and library deposit, if such use conforms to the customary and usual practice of the Sub-Licensee. Reproductions in printed or electronic form of Academic Works containing Digital Maps only may be provided to sponsors of such Academic Works. Reproductions in electronic form of Academic Works containing Digital Data may only be provided to sponsors of such Academic Works where such sponsor holds a licence from the Licensor to use Digital Data. Reproductions in printed form of Academic Works containing Digital Data may be provided to sponsors of such Academic Works. In each case, each item shall carry the following copyright notice "© Crown Copyright/database right 20(yy). An Ordnance Survey/ (Datacentre) supplied service". Sponsors of Academic Works may not use any Reproductions provided to them under the provisions of this Clause 3.1.6 for any Commercial Use; and

3.1.7 incorporate parts of the Digital Maps in printed and electronic conference papers, articles, articles published in a journal publication and poster sessions;

3.1.8 provide printed or electronic copies of the Licensed Work and Digital Maps at the request of individual Authorised Users;

3.1.9 supply to an authorised user of another Institution library (whether by post, fax or secure electronic transmission, using Ariel or its equivalent, whereby the electronic file is deleted immediately after printing) a single paper copy of parts of the Licensed Work. Each copy shall carry the following copyright notice "© Crown Copyright/database right 20(yy). An Ordnance Survey/(Datacentre) supplied service";

3.1.10 display, download and print parts of the Licensed Work and Digital Maps for the purpose of promotion of the Licensed Work, testing of the Licensed Work and Digital Maps, or for training Authorised Users;

3.1.11 publicly display or publicly perform parts of the Licensed Work and Digital Maps as part of a presentation at a seminar, conference, or workshop, or other such similar activity;

3.1.12 make such copies of training material and network on a Secure Network such training material as may be required for the purpose of using the Licensed Work and Digital Maps in accordance with this Sub-Licence.

3.1.13 use Digital Maps in brochures, leaflets and flyers for use by the Sub-Licensee;

3.1.14 publish Digital Maps on the website of the Sub-Licensee or websites created by Authorised Users as part of a course or project;

3.1.15 print and use copies of the Licensed Work and Digital Maps as part of a Post Graduate Certificate in Education course in schools.

3.2 Nothing in this Sub-Licence shall constitute a waiver of any statutory right available and held by the Sub-Licensee and/or Authorised Users from time to time under the Copyright, Designs and Patents Act 1988 or any amending legislation.

## 4. RESTRICTIONS

4.1 Save as provided herein, the Sub-Licensee and Authorised Users may not:

4.1.1 sell, resell the Licensed Work and/or Digital Maps unless the Sub-Licensee has been granted prior written consent by Ordnance Survey to do so;

4.1.2 redistribute, publish or otherwise make the information contained in the Licensed Work and Digital Maps available in any manner or on any media other than that allowed under this Agreement;

4.1.3 remove, obscure or modify copyright notices, text acknowledgments or other means of identification or disclaimers as they appear;

4.1.4 make printed or electronic copies of more than 10% of the whole of Land-Line Plus® for any purpose;

4.1.5 save in relation to Clauses 3.1.10, 3.1.11 and 3.1.14, display or distribute any part of the Licensed Work and Digital Maps on any electronic network, including without limitation the Internet and the World Wide Web, and any other distribution medium now in existence or hereinafter created, other than on a Secure Network;

4.1.6 save in relation to Clauses 3.1.6, 3.1.9, 3.1.14 and 3.1.15, permit or allow anyone other than Authorised Users to access or use the Licensed Work and Digital Maps;

4.1.7 use all or any part of the Licensed Work and Digital Maps for any Commercial Use or for any purpose other than Educational Purposes;

4.1.8 save in relation to Clause 3.1.9, permit the Licensed Work to be electronically transmitted to any other recipient except where the recipient is an Authorised User under the terms of a valid Ordnance Survey Data Sub-Licence Agreement.

4.2 This Clause shall survive termination of this Sub-Licence for any reason.

## 5. RESPONSIBILITIES OF THE SUB-LICENSEE

5.1 The Sub-Licensee will:

5.1.1 issue passwords or other access information only to Authorised Users and use all reasonable efforts to ensure that Authorised Users do not divulge their passwords or other access information to any third party;

5.1.2 use all reasonable efforts, including without limitation by use of Secure Authentication, to ensure that only Authorised Users are permitted access to the Licensed Work and Digital Maps;

5.1.3 use all reasonable efforts to ensure that all Authorised Users are made aware of and undertake to abide by the terms of this Sub-Licence;

5.1.4 use all reasonable efforts to monitor compliance with the terms of this Sub-Licence and notify Ordnance Survey and HEFCE (including any Datacentre acting on behalf of HEFCE in the provision of the Service) immediately and provide full particulars on becoming aware of any of the following (a) any unauthorised access to or use of the Licensed Work and Digital Maps or unauthorised use of any of the Sub-Licensee's password(s); or (b) any breach by an Authorised User of the terms of this Sub-Licence. Upon becoming aware of any breach of the terms of this Sub-Licence the Sub-Licensee further agrees promptly to fully investigate and initiate disciplinary procedures in accordance with the Sub-Licensee's standard practice and use all reasonable effort to ensure that such activity ceases and to prevent any recurrence;

5.1.5 comply with all computer security procedures required by Ordnance Survey, JISC Collections and/or the Datacentre and take all reasonable steps to ensure the security of the Licensed Work and Digital Maps in accordance with the Security Requirements set out in Appendix 3 hereto;

5.1.6 where IP addresses are used as the method of Secure Authentication, provide lists of valid IP addresses to JISC and update those lists on a regular basis the frequency of which will be agreed by the parties from time to time;

5.1.7 make available to Ordnance Survey on request such information as Ordnance Survey may reasonably require to use to better understand the type and nature of the specific Educational Purposes carried out by the Sub-Licensee and the Authorised Users using the Licensed Work and Digital Maps. In particular but without prejudice to the generality of the foregoing, Ordnance Survey may use examples of such use for the preparation of case studies for marketing and developing the Licensed Work and Digital Maps.

## 6. RESPONSIBILITIES OF JISC COLLECTIONS

6.1 JISC Collections shall use all reasonable efforts to ensure access and use of the Licensed Work in accordance with the provisions as laid down in this Sub-Licence.

6.2 JISC Collections shall use all reasonable efforts to cause customer support services to be provided to the Sub-Licensee and to Authorised Users.

## 7. USAGE DATA

7.1 The parties shall co-operate in gathering any data on usage of the Licensed Work that is available to them during the term of this Sub-Licence, and shall provide such data to each other upon request.

## 8. FEE

8.1 The Sub-Licensee shall pay an annual fee to JISC Collections for the Sub-Licence in the amount and upon terms set out in Appendix 1.

## 9. TERM AND TERMINATION

9.1 The term of this Sub-Licence will commence upon the date of signature and will remain in full force and effect until 31st July 2009, unless terminated earlier as provided for in this Clause 9.

9.2 Either party may terminate this Sub-Licence at any time on the material breach or repeated other breaches by the other of any obligation on its part under this Sub-Licence by serving a written notice on the other identifying the nature of the breach. The termination will become effective thirty days after receipt of the written notice unless during the relevant period of thirty (30) days the defaulting party remedies the breach.

9.3 Notwithstanding anything to the contrary herein, this Sub-Licence will automatically terminate if the Ordnance Survey Data Supply and Licence Agreement between Ordnance Survey and JISC Collections terminates for any reason. JISC Collections shall make reasonable endeavours not to cause the Ordnance Survey Data Supply and Licence Agreement between Ordnance Survey and JISC Collections to terminate.

9.4 Further, notwithstanding anything to the contrary herein, upon a breach by the Sub-Licensee, on-line access to the Licensed Work and Digital Maps shall be terminated.

9.5 Upon termination of this Sub-Licence, copies of parts of Digital Maps made by the Sub-Licensee or Authorised Users may be retained and used subject to the terms of Clauses 3 and 4 of this Sub-Licence, which terms shall survive any termination of this Sub-Licence.

9.6 Upon termination of this Sub-Licence, and only if requested by JISC Collections, all electronic copies of the Digital Data within the possession or control of the Sub-Licensee shall be deleted within thirty (30) days of such request and the Sub-Licensee shall confirm such deletion to JISC Collections. The Sub-Licensee shall also use its reasonable endeavours to procure that any electronic copies of the Digital Data within the possession or control of the Authorised Users are also destroyed within the said period of thirty (30) days. For the avoidance of doubt, printed copies of the Digital Data may be retained and used subject to the terms of Clauses 3 and 4 of this Sub-Licence

## 10. ACKNOWLEDGEMENT AND PROTECTION OF INTELLECTUAL PROPERTY RIGHTS

10.1 The Sub-Licensee acknowledges that all Intellectual Property Rights in the Licensed Work and Digital Maps (save for the Intellectual Property Rights in the software used by a Datacentre in the creation of Digital Maps and any Intellectual Property Rights in any Service or Open Access Service provided by a Datacentre other than the Licensed Work and Digital Maps themselves) are the sole and exclusive property of Ordnance Survey or are duly licensed to Ordnance Survey and that this Sub-Licence does not assign or transfer to the Sub-Licensee any right, title or interest therein except for the right to use the Licensed Work and Digital Maps in accordance with the terms and conditions of this Sub-Licence.

## 11. REPRESENTATIONS, WARRANTIES AND INDEMNIFICATION

11.1 The Sub-Licensee represents and warrants that it has sufficient authority to enter into and perform its obligations under this Sub-Licence.

11.2 JISC Collections makes no representation or warranty, and expressly disclaims any liability with respect to the content of the Licensed Work and Digital Maps, including but not limited to errors or omissions contained therein, libel, infringement of rights of publicity, privacy, trademark rights, moral rights, or the disclosure of confidential information. It is expressly agreed that any use by the Sub-Licensee or by any Authorised Users of the Licensed Work and Digital Maps is at the Sub-Licensee's sole risk.

11.3 The Sub-Licensee agrees to notify Ordnance Survey and JISC Collections including the Datacentre acting on behalf of JISC Collections immediately and provide full particulars in the event that it becomes aware of any actual or threatened claims by any third party in connection with any works contained in the Licensed Work and Digital Maps. It is expressly agreed that upon such notification, or if Ordnance Survey becomes aware of such a claim from other sources, Ordnance Survey may remove such work(s) from the Licensed Work and Digital Maps. At the request of Ordnance Survey, the Sub-Licensee will remove such work(s) from any copies of the Licensed Work and Digital Maps maintained by the Sub-Licensee and shall make reasonable efforts to remove such works from any copies of the Licensed Work and Digital Maps in the possession or control of the Authorised Users. Failure to report knowledge of any actual or threatened claim by any third party shall be deemed a material breach of this Agreement.

11.4 To the extent permitted by law, JISC Collections shall not be liable to the Sub-Licensee for any loss or damage including any loss of profits, goodwill, contract or any indirect or consequential loss including loss or damage suffered by the Sub-Licensee as a result of an action brought by a third party.

11.5 The Sub-Licensee shall fully indemnify JISC Collections against all claims, demands, actions, costs, expenses (including but not limited to full legal costs and disbursements), losses and damages arising from or incurred by reason of any default, act or omission of the Sub-Licensee or its Authorised Users connected with any infringement or alleged infringement (including but not limited to the defence of such alleged infringements) of any Intellectual Property Rights enforceable in the United Kingdom in connection with the subject matter of this Agreement.

11.6 The Licensed Work and Digital Maps is provided on an "as is" basis, and JISC Collections disclaims any and all warranties, conditions, or representations (express, implied, oral or written), relating to the Licensed Work and Digital Maps or any part thereof, including, without limitation, any and all implied warranties of quality, performance, merchantability or fitness for a particular purpose. JISC Collections further expressly disclaims any warranty or representation to Authorised Users, or to any third party. JISC Collections accepts no liability for loss suffered or incurred by the Sub-Licensee or Authorised Users as a result of their reliance on the Licensed Work and Digital Maps.

11.7 The Sub-Licensee represents to JISC Collections that its computer system through which the Licensed Work and Digital Maps will be used is configured, and procedures are in place, to prohibit access to the Licensed Work and Digital Maps by any person other than an Authorised User; that it shall inform Authorised Users about the conditions of use of the Licensed Work and Digital Maps; and that during the term of this Sub-Licence, the Sub-Licensee will continue to make all reasonable efforts to bar non-permitted access and to convey appropriate use information to its Authorised Users.

## 12. FORCE MAJEURE

12.1 Either party's failure to perform any term or condition of this Sub-Licence as a result of circumstances beyond the control of the relevant party (including without limitation, war, strikes, flood, governmental restrictions, and power, telecommunications or Internet failures or damages to or destruction of any network facilities) ("Force Majeure") shall not be deemed to be, or to give rise to, a breach of this Sub-Licence.

12.2 If either party to this Sub-Licence is prevented or delayed in the performance of any of its obligations under this Sub-Licence by Force Majeure and if such party gives written notice thereof to the other party specifying the matters constituting Force Majeure together with such evidence as it reasonably can give and specifying the period for which it is estimated that such prevention or delay will continue, then the party in question shall be excused the performance or the punctual performance as the case may be as from the date of such notice for so long as such cause of prevention or delay shall continue.

## 13. ASSIGNMENT

13.1 Neither this Sub-Licence nor any of the rights and obligations under it may be assigned or sub-licensed by the Sub-Licensee without obtaining the prior written consent of JISC Collections, such consent not to be unreasonably withheld or delayed. In any permitted assignment, the assignor shall procure and ensure that the assignee shall assume all rights and obligations of the assignor under this Sub-Licence and agrees to be bound to all the terms of this Sub-Licence.

13.2 Notwithstanding the provisions of Clause 13.1, this Sub-Licence may not be assigned by the Sub-Licensee to any organisation that is not an Institution.

## 14. FREEDOM OF INFORMATION

14.1 For the purposes of Clause 14.3 below, "Disclosing Party" means the party to this Agreement from whom the relevant information originated. "Receiving Party" means the party in receipt of the relevant information and which is asked to disclose the relevant information under the FOIA.

14.2 Each party shall, both during and following expiry of this Agreement, use all reasonable endeavours to assist the other party to comply with its obligations (if any) under the FOIA.

14.3 Pursuant to Clause 14.2, but without prejudice to the generality of the same, a Receiving Party shall:

14.3.1 promptly inform the Disclosing Party of any request for information received under the FOIA and the nature of the information being sought;

14.3.2 not disclose, release information or otherwise respond to a request for information without prior reference to, discussion with and authorisation from the Disclosing Party;

14.3.3 consider and apply all lawful exemptions provided under the FOIA to withhold information sought under a request for information

## 15. GOVERNING LAW AND DISPUTE RESOLUTION

15.1 This Sub-Licence shall be governed by and construed in accordance with English law and the parties irrevocably agree that any dispute arising out of or in connection with this Sub-Licence will be subject to and within the jurisdiction of the English courts.

15.2 Where the parties agree that a dispute arising out of or in connection with this Sub-Licence would best be resolved by the decision of an expert, they will agree upon the nature of the expert required and together appoint a suitable expert by agreement. In default of agreement upon whom to appoint as a suitable expert, such expert shall upon the request of either party be appointed by the Chair for the time being of the Committee of Vice-Chancellors and Principals.

15.3 Any person to whom a reference is made under Clause 15.2 shall act as expert and not as an arbitrator and his decision (which shall be given by him in writing and shall state the reasons for his decision) shall be final and binding on the parties except in the case of manifest error or fraud.

15.4 Each party shall provide the expert with such information and documentation as he may reasonably require for the purposes of his decision.

15.5 The costs of the expert shall be borne by the parties in such proportions as the expert may determine to be fair and reasonable in all circumstances or, if no determination is made by the expert, by the parties in equal proportions.

## 16. NOTICES

16.1 All notices required to be given under this Sub-Licence shall be given in writing in English and sent by electronic mail, fax or first class registered or recorded delivery to the relevant addressee at its address set out below, or to such other address as may be notified by either party to the other from time to time under this Sub-Licence, and all such notices shall be deemed to have been received (a) 24 hours after successful transmission in the case of electronic mail or fax; (b) fourteen (14) days after the date of posting in the case of first class registered or recorded delivery:

if to JISC Collections:
Lorraine Estelle
CEO
Jic Collections
Brettenham House
5 Lancaster Place
London
WC2E 7EN
Email: [l.estelle@jisc.ac.uk](mailto:l.estelle@jisc.ac.uk)

if to Ordnance Survey:
External Research Manager
Research C530
Ordnance Survey
Romsey Road
Southampton
SO16 4GU
Fax: 023 8030 5072
Email: [universityenquiries@ordnancesurvey.co.uk](mailto:universityenquiries@ordnancesurvey.co.uk)

if to the Sub-Licensee: [As stated in the Acceptance of Sub-Licence Form]

if to Datacentre:
Manager, Research and Geodata Services
EDINA
Causewayside House,
160 Causewayside,
Edinburgh,
Scotland,
United Kingdom
EH9 1PR
Fax: (0)131 650 3308

## 17. GENERAL

17.1 This Sub-Licence and its Appendices constitute the entire agreement between the parties relating to the Licensed Work and supersede all prior communications, understandings and agreements (whether written or oral) relating to its subject matter and may not be amended or modified except by agreement of both parties in writing.

17.2 The Appendices shall have the same force and effect as if expressly set in the body of this Sub-Licence and any reference to this Sub-Licence shall include the Schedules.

17.3 Ordnance Survey may enforce the terms of this Agreement subject to and in accordance with the terms of this Sub-Licence and the provisions of the Contract (Rights of Third Parties) Act 1999, as amended or substituted from time to time.

17.4 The invalidity or unenforceability of any provision of this Sub-Licence shall not affect the continuation in force of the remainder of this Sub-Licence.

17.5 The rights of the parties arising under this Sub-Licence shall not be waived except in writing. Any waiver of any of a party's rights under this Sub-Licence or of any breach of this Sub-Licence by the other party shall not be construed as a waiver of any other rights or of any other or further breach. Failure by either party to exercise or enforce any rights conferred upon it by this Sub-Licence shall not be deemed to be a waiver of any such rights or operate so as to bar the exercise or enforcement thereof at any subsequent time or times.

## APPENDIX 1 - FEE AND PAYMENT TERMS

1. By returning a signed copy of this Sub-Licence, the Sub-Licensee accepts that it must pay the total Fee applicable to their institution over the whole term of this Sub-Licence. JISC Collections through a JISC Collections-authorised agent will invoice the Sub-Licensee annually for the applicable Fee as set out below. All Fees are exclusive of VAT, which will be payable where applicable.

Sub-Licensee Charges for higher education institutions and research councils:

| **JISC Band** | **Annual Subscription Fees (excl. VAT)* 1st August 2007 to 31st July 2008** | **Annual Subscription Fees (excl. VAT)* 1st August 2008 to 31st July 2009** |
| --- | --- | --- |
| A - B | £6,471 | £6,924 |
| C - D | £5,449 | £5,831 |
| E - F | £4,428 | £4,738 |
| G - J | £2,384 | £2,551 |

Sub-Licensee Charges for further education institutions:

| **JISC Band** | **Annual Subscription Fees (excl. VAT)* 1st August 2007 to 31st July 2008** | **Annual Subscription Fees (excl. VAT)* 1st August 2008 to 31st July 2009** |
| --- | --- | --- |
| D - F | £300 | £300 |
| G - H | £275 | £275 |
| I - J | £250 | £250 |

Invoices shall be due and payable no later than thirty (30) days after being issued by JISC Collections or a JISC Collection-authorised agent.

**In addition, the Sub-Licensee accepts that it must pay an annual Crown Copyright Fee of £300 + VAT per annum for HE and Research Councils, for FE the cost is £145 + VAT and for 6th Form Colleges, £88 + VAT which shall be payable directly to Ordnance Survey. An invoice will be issued by Ordnance Survey directly to the Sub-Licensee.**

A copyright licence application form is available from the Ordnance Survey Copyright web pages at: <http://www.ordnancesurvey.co.uk/oswebsite/education/pdf/L0120_M_without_watermark.pdf>

Supporting information can be found at:

<http://www.ordnancesurvey.co.uk/oswebsite/education/pdf/L0167_M.pdf>

Completed licences should be sent to Ordnance Survey:

Customer Service Centre
Ordnance Survey
Romsey Road
Southampton
SO16 4GU
Telephone: 023 8030 5030
Fax: 023 8079 2615

## APPENDIX 2: LICENSED WORK

The Licensed Work consists of the following products:

- OS MasterMap®- Topographic Layer (this product is not currently available but it is the intention that it will be by September 2007)
- OS Mastermap®- Integrated Transport Network Layer (this product is not currently available but it is the intention that it will be by September 2007)
- Land-Line.Plus®
- 1:10 000 Scale Colour Raster
- 1:25 000 Scale Colour Raster
- 1:50 000 Scale Colour Raster
- 1:50 000 Scale Gazetteer
- Strategi®
- Meridian 2™
- Code-Point®
- Code-Point® with polygons
- Land-Form PROFILE®-contours & Digital Terrain Mapping
- Land-Form PANORAMA™ - contours & Digital Terrain Mapping
- Boundary-Line™

## APPENDIX 3 - SECURITY REQUIREMENTS

The security requirements for the supply by a Service and use of the Licensed Work and Digital Maps in Authorised Institutions under the terms of this Agreement are set out below.

### Overall Objectives

These are as follows, save as provided herein:

- To ensure that only Authorised Users are allowed to use the Licensed Work and Digital Maps.
- To ensure that Authorised Users are aware of the terms of use of the Licensed Work and Digital Maps
- To ensure that use of the Licensed Work and Digital Maps can be monitored traced and analysed
- To ensure that outputs generated from the Licensed Work and Digital Maps can be identified.

### 1. HEFCE's Responsibilities

1.1 HEFCE will ensure that the Datacentre will fulfil the security objectives set out in paragraph 1 above.

1.2 HEFCE will ensure that the Datacentre appoints a Responsible Officer for the security of the Licensed Work and Digital Maps at the Datacentre and for ensuring that the defined security procedures consistent with meeting the objectives at paragraph 1 are implemented. The name of the Datacentre Responsible Officer will be notified to HEFCE together with a nominated deputy. If the individual fulfilling this role changes, HEFCE will be notified as soon as possible.

### 2. Datacentre's Security Role

The Datacentre will, save as provided herein:

- Establish roles and responsibilities within the Sub-Licensee to apply and to keep records of the individuals fulfilling these roles.
- Implement an incidents procedure in the event of mis-use of the Licensed Work and Digital Maps being discovered.

### 3. Subscription

3.1 A Sub-Licensee wishing to access and use the Licensed Work and Digital Maps will be required to:

- Formally appoint and identify a Site Representative(s) who will be responsible for the Licensed Work and Digital Maps
- Formally appoint and identify a Responsible Officer for Data Security
- Formally appoint and identify a Deputy Responsible Officer for Data Security

3.2 The roles of the Site Representative(s) and Security Officers will be as follows:

- **Site Representative(s)** - will be the contact for the Service within the Authorised Institution. They will be responsible for countersigning any documentation relating to user activities, where required. They will also be responsible for general promotion of the service and support for the service relating to local infrastructure and procedures.

It may be convenient for some Authorised Institutions to have more than one representative, particularly where the Authorised Institution is located across more than one site. In such cases however each Site Representative will need to be nominated formally by the Authorised Institution. The maximum number of Site Representatives is not expected to exceed 5.

- **Responsible Officer for Data Security (and Deputy)** - will take responsibility for ensuring the observance of the conditions for use of the Licensed Work and Digital Maps under the terms of this Agreement by all Authorised Users of their Authorised Institution. They have a duty to report to the Datacentre any suspected security weaknesses of the Service provided by that Datacentre and to advise the Datacentre immediately of any actual incidences of misuse of the Licensed Work and Digital Maps.

3.3 The Datacentre will maintain a current list of Site Representatives and Responsible Security Officers (and Deputies) together with their signatures and contact addresses. The Datacentre will provide this list to Ordnance Survey upon request.

### 4. User Registration

4.1 Authorised Institution will be required to:

- Ensure that only Authorised Users under the terms of this Agreement have access to the Licensed Work and Digital Maps
- Require Authorised Users to hold personal authentication credentials e.g. an individual Athens account or local institution login details which are integrated into the UK Access Management Federation or another authentication system recognised and supported by JISC.
- Where class registration is needed, require each member of the class to sign a class registration form, and to identify the Athens accounts to be used for class registration. Note that Class registration is not possible where an institution implements authentication and authorisation systems involving Athens Devolved Authentication or UK Access Management Federation.
- Require the class lecturer to be the Responsible Officer for use of the Licensed Work and Digital Maps under Class registration
- Identify which Athens account is to be allocated to the class lecturer nominated as the Responsible Officer for class registration
- Require each Class registration to be terminated after 3 months

4.2 Authorised Users will be required to personally register for access to the Licensed Work and Digital Maps using a web-based form.

4.3 Data collected by the Datacentre under the registration system is done so in accordance with the Data Protection Act. Users will be made aware that information is stored about them and their usage of the Licensed Work and Digital Maps.

4.4 On receipt of a personal user registration application or class registration form, the Datacentre will check the details submitted and record users details onto a master User Database.

4.5 Registrations contained in the User Database which are not used to access the Service for a period of 12 months will be deactivated by the Datacentre.

### 5. Training and Information

5.1 The Datacentre will run a scheme of training for Site Representatives and Responsible Officers for Security at Authorised Institutions. The training will provide information on Authorised User Registration procedures, security of the Licensed Work and Digital Maps, the usage of Licensed Work and Digital Maps permitted under the Agreement and copyright arrangements.

5.2 The Datacentre will promote the availability of these courses widely across the higher and further education communities and the research councils. This activity will be ongoing throughout the period the Datacentre is contracted to manage the secure distribution and use of the Licensed Work and Digital Maps. Authorised Institutions are strongly encouraged to nominate relevant staff to attend, and to ensure that staff are familiar with the terms and conditions of use of the Service, Licensed Work and Digital Maps.

5.3 In order to re-enforce good practice amongst Authorised Users of the Licensed Work and Digital Maps at the Authorised Institution, the Datacentre will develop and implement procedures as part of their delivery systems and / or within the Authorised Institution that: -

- Display the copyright terms and conditions that apply to use of the Licensed Work and Digital Maps whenever an Authorised User logs on to the Service.
- Ensures that Authorised Users are required to read and accept the terms of Use for the Licensed Work and Digital Maps as part of the registration service.
- Provide ready access to a Terms of Use summary for all Authorised Users accessing the Licensed Work and Digital Maps.
- Ensure that a copy of the Terms of Use summary is supplied with all copies of the Licensed Work supplied to an Authorised Institution.

### 6. Access Control to the Service

6.1 The Datacentre will, save as provided herein:

- Ensure only Authorised Users can have access to the Licensed Work and Digital Maps.
- Ensure that Authorised Users are only given access to those parts of the Licensed Work and Digital Maps and the Service to which they are eligible.

6.2 Access to the Licensed Work and the Digital Maps will be authorised by the Athens Access Management System or the UK Access Management Federation. The Authorised Institution is required to record authorisation for its users using Athens or their local authentication and authorisation system. Individual Authorised Users will also be required to register with the Datacentre in order to access the Licensed Work and Digital Maps.

6.3 On having been given access to the Service, the system is required to display a document setting out copyright terms and conditions for use of the Licensed Work and Digital Maps. The Authorised User will be required to indicate acceptance of these terms and conditions before being given access to the Licensed Work and Digital Maps.

6.4 The Datacentre access system will be required to check that the Authorised User has correctly logged into the Service whenever that Service is accessed. If the check is not positive then the Authorised User will be prompted to provide their username and password which will then be authenticated in accordance with paragraph 6.2 above. This ongoing checking process is required to ensure that bookmarking the Datacentre's Service does not by-pass the Authorised User authentication access controls.

6.5 The Datacentre access system will be required to provide specific access controls to each of the levels of Service provided including:

- The Licensed Work and Digital Maps provided under the terms of the Sub-Licence
- Support information provided for the benefit of Site Representatives and Responsible Officers for security of the Licensed Work and Digital Maps
- The types of service available from the Licensed Work and Digital Maps such as:
  - Data download.
  - Digital Maps generated from the Licensed Work and Digital Maps.

6.6 The Datacentre access system will be required to provide an automatic cut-out that will disconnect the Authorised User from access to the Licensed Work if they do not interact with the Service for a period of 30 minutes. Where such a cut-out occurs, before the Service can be accessed again, the system will require user details to be re-authenticated in accordance with paragraphs 6.2 and 6.3 above.

6.7 Downloading data.

The Datacentre will:

- Ensure that only the Authorised User who has requested the Licensed Work and Digital Maps in data form is able to download it.
- Ensure that these procedures can offer a secure environment for data downloaded through HTTP including a time limit for the period that the data is available for HTTP download. This period must not exceed 3 days.

### 7. Identifying maps and data

7.1 The Datacentre will:

- Watermark all screen images of mapping generated from the Licensed Work and Digital Maps and annotate the image with a standard Crown copyright statement that will appear along the bottom edge of the image.
- Watermark all hard copy map images printed from the Licensed Work and Digital Maps and display a Crown copyright acknowledgement.
- On all hard copy map images printed from the Licensed Work and Digital Maps include the Authorised User's name and the date of image production.
- Identify the Datacentre as the source for any of the Licensed Work and Digital Maps products supplied in data form to Authorised Users along with the Authorised User name and the date the Licensed Work and Digital Maps was requested for downloading. This information is to be written into each data file where technically possible at reasonable cost..

### 8. Logging user activity

8.1 The Datacentre will log and monitor the use of the Licensed Work and Digital Maps by Authorised Users as follows:

- To record individual Authorised User activity for audit purposes by the Datacentre, and/or Ordnance Survey
- To identify individual Authorised User activity by hard copy map extracts and data download
- To make Site Representatives aware of the pattern of usage within the Authorised Institution
- To make Authorised Users aware of any unauthorised use of their Athens account or personal authentication credentials
- To make the Datacentre aware of any unusual patterns of usage of the Licensed Work and Digital Maps either by individual Authorised Users or by Authorised Institutions
- Save as provided in Clause 5.2.8, to record the transfer of data downloaded to an Authorised User from one Authorised Institution to another Authorised User at another Authorised Institution

8.2 All logs of use of the Licensed Work and Digital Maps will be retained and made accessible by the Datacentre to Ordnance Survey and/or HEFCE and/or Site Representatives for a rolling period of 5 years. In accordance with the Data Protection Act users will be notified that their use of the Licensed Work and Digital Maps is monitored and recorded.

### 9. Incident Procedures

9.1 Reporting of Incidents

9.1.1 Where a suspected incident of misuse of the Licensed Work and Digital Maps is detected by Ordnance Survey and/or HEFCE and/or the Datacentre and/or the Sub-Licensee and/or by an Authorised User, details of the incident will be relayed immediately to the Datacentre. The Datacentre will in turn notify the relevant Authorised Institution, HEFCE and Ordnance Survey.

9.1.2 In all cases the Datacentre will document the details of the incident in a Security Incident Log. As the investigation into the incident proceeds, this log will be updated accordingly. Incidents will be classified under "User" or "Institution" in accordance with the definitions in paragraph 10.2 below. The content of the Security incident log will be made available at all times to Ordnance Survey and/or HEFCE. The Security Incident Log will be retained by the Datacentre for a period of 3 years beyond the cessation of service operation by the Datacentre.

9.1.3 Classification of Security Incidents

The Datacentre will be required to identify an incident under one of two categories as follows:

- **User-related Incident** - An isolated case in which some aspect of licence conditions were not fulfilled by an Authorised User that raises doubts as to the adequacy of the measures to protect the availability, integrity, correct usage or supply of the Licensed Work and Digital Maps to Authorised Users only e.g. an isolated incident of misuse of the Licensed Work and Digital Maps due to an Authorised User's misinterpretation of the terms of the Sub-Licence agreement between JISC Collections and the Authorised Institution; a breach of password security by an individual user leading to access and use by unauthorised individuals.
- **Institutional-level Incident** - A failure to comply with the Sub-Licence conditions of the Licensed Work and Digital Maps that raises doubts as to the adequacy of measures within an Authorised Institution to protect the availability, integrity, correct usage or supply of elements of the Licensed Work and Digital Maps to Authorised Users only e.g. a systematic failure to acquaint users of the Licensed Work and Digital Maps with the terms of use and an environment which fails to provide the necessary checks required under the terms of the licence for supply of the Licensed Work and Digital Maps; systematic failure to manage local authentication and authorisation procedures appropriately; incidents of access and use by unauthorised individuals.

### 10.Resolution of Incidents

#### User-related Incident

- When a user-related security incident is identified, the Responsible Officer for Data Security at the relevant Authorised Institution will report the details to the Datacentre immediately where it will be logged in the Security Incident Log.
- The Datacentre will report details of the incident to Ordnance Survey, HEFCE and JISC Collections when there is sufficient information about the incident to report.
- If appropriate, the Datacentre will remove access to the Licensed Work and Digital Maps from one or more Authorised Users to prevent further security incidents or exacerbation of an incident already identified.
- The Responsible Officer for Data Security at the Authorised Institution will, in consultation with the Site Representative and the Datacentre as necessary, draw up and initiate a corrective action plan within 10 working days of the incident being identified. The Datacentre will consult with HEFCE and Ordnance Survey to agree appropriate corrective action. HEFCE and Ordnance Survey will agree what action is required within 10 working days of being notified by the Datacentre so that the Datacentre can liaise with the Authorised Institution to implement the corrective action plan.
- The corrective action plan will include a timetable for implementation that will not normally exceed 6 working weeks from the date the incident was identified.
- The Datacentre will insist on regular reports from the Responsible Officer until the action plan to rectify the incident has been completed.
- On completion, the Responsible Officer will notify the Datacentre and, on agreement from HEFCE and Ordnance Survey, the incident will be recorded as closed in the Security Incident log. A copy of the full Incident Log entry will be sent to Ordnance Survey and HEFCE for information.
- When the Responsible Officer for Data Security at a Authorised Institution is unable to draw up a practical action plan to resolve the security incident to the satisfaction of the Datacentre, the Datacentre will immediately inform Ordnance Survey and HEFCE.
- Ordnance Survey and the Datacentre will then provide the Responsible Officer and the relevant Authorised Institution with direct guidance to resolve the incident satisfactorily.
- Should the incident not be resolved satisfactorily, and within the agreed timescale, with direct guidance from Ordnance Survey and the Datacentre, further action may be taken against the Authorised Institution as detailed under an Institutional-level Incident.

#### Institutional-level Incident

- On identifying an institutional-level incident, the Responsible Officer for Security at the relevant Authorised Institution will inform the Site Representative (and vice-versa) and notify the Datacentre immediately.
- When an institutional-level incident is raised with the Datacentre whether by a Responsible Officer for Security at a Authorised Institution or from another source, the Datacentre will immediately record the circumstances in the Security Incident Log and inform Ordnance Survey and HEFCE.
- The Datacentre will request that the Authorised Institution Institution take significant remedial action and give a timetable for its implementation. Both the remedial action and the timetable for its implementation shall be agreed with the Datacentre and Ordnance Survey and must not exceed six working weeks duration commencing from the date the incident was first identified.
- The Datacentre will instruct the Responsible Officer for Security to provide weekly progress reports about the implementation of the remedial action plan.
- Once the Responsible Officer for Security has reported that the problem has been resolved (assuming that it is within the timetable period) the Datacentre will require documentary evidence that satisfactorily demonstrates this resolution.
- If the Datacentre or Ordnance Survey are not satisfied with this evidence however, the Datacentre will advise the Authorised Institution at senior management level and require assistance in pursuing any further investigation to establish that the problem has been properly resolved.
- Subject to this assistance being forthcoming and the necessary evidence of corrective action being provided, the Datacentre may, subject to agreement from Ordnance Survey, declare the incident closed.
- The Security Incident Log entry will be completed by the Datacentre to record the sequence of actions taken and the date the incident was closed.
- If however the problem is not resolved in a timely fashion and to the satisfaction of the Datacentre, the Datacentre will recommend to Ordnance Survey and HEFCE that the offending Authorised Institution's right to access the Licensed Work and Digital Maps be suspended. Examples of where this action will be applicable are as follows:
  - When significant abuse has actually taken place (rather than a weakness in procedures that creates the potential for abuse being identified).
  - Where in the opinion of the Datacentre, having consulted with Ordnance Survey, the incident represents an unacceptable risk to the Licensed Work and Digital Maps and will take significant time to resolve.
  - Where the Authorised Institution has failed to provide clear evidence of adequate remedial action within an agreed period of time.
  - Where there is evidence of a repeated lack of commitment within the Authorised Institution to apply agreed security procedures.Where following an on site examination by the Datacentre, the Authorised Institution fails to show satisfactory evidence that the reasons for the institutional-level security incident have been properly resolved.

If the Datacentre's recommendation for suspension is agreed by Ordnance Survey and HEFCE, the Datacentre will take the following action within two working weeks:

- Immediately terminate all access to the Licensed Work and Digital Maps or related Services provided by the Datacentre to all Authorised Users at the relevant Authorised Institution.
- Immediately, ensure that the offending Authorised Institution has no access to the Licensed Work or related Services from other Authorised Institutions or their Authorised Users.
- Place a moratorium on the continued use of Licensed Work and Digital Maps or outputs from the Licensed Work and Digital Maps already held within the offending Authorised Institution and ensure that Authorised Users within that Authorised Institution are aware that any continued use would be in breach of the terms of this Agreement. In this regard, the Datacentre will provide additional support to the Authorised Institution Site Representative and Responsible Officer for Security.
- Set a suspension period for the Authorised Institution and notify the Site Representative and Responsible Officer for Security accordingly.

Suspension Period and Re-instatement

- During the period of suspension, the Datacentre will support the Responsible Officer for Security and the Site Representative at the offending institution to implement the agreed action plan to resolve the security problem. The Datacentre will instruct the Responsible Officer for Security and the Site Representative to provide weekly reports on implementation progress.
- One week prior to the end of the agreed suspension period, the Datacentre will review the corrective action taken and decide whether the security problem has been resolved satisfactorily. If this is the case, it will advise Ordnance Survey and HEFCE accordingly and recommend reinstatement of the Authorised Institution. Subject to Ordnance Survey and the HEFCE' agreement, the Datacentre can initiate re-instatement action having recorded the decision in the Security Incident Log and the planned re-instatement date.
- Ordnance Survey may, prior to agreeing to re-instatement, request that the Datacentre carry out a formal site audit of the Authorised Institution and produce a written report of its findings. Copies of this report will be sent to Ordnance Survey and HEFCE to inform their decision on re-instatement.

Possible Termination

- If the offending Authorised Institution is found not to have resolved the security problem to the satisfaction of the Datacentre, Ordnance Survey and HEFCE, then the Authorised Institution will be deemed to have terminated its right to access the Licensed Work and Digital Maps under the terms of the Ordnance Survey Data Sub-Licence Agreement.
- In the event of the termination of a Authorised Institution's licence to use the Licensed Work, HEFCE and Ordnance Survey, with assistance from the Datacentre, will be required to take the following action:
  - Advise the Authorised Institution formally in writing that its access to the Licensed Work and Digital Maps has been terminated and the reasons for this decision.
  - Advise the Authorised Institution within 1 week (and supervise its compliance) to destroy all copies of the Digital Data held on the Authorised Institution's systems.
  - Advise the Authorised Institution within 1 week (and supervise its compliance) to instruct all Authorised Users of the Digital Data within its establishment to delete all electronic copies of the Digital Data they may hold in their personal files within 3 weeks. Failure to do so will mean that Authorised Users are in breach of Crown Copyright.
  - Advise the Authorised Institution (and supervise its compliance) to instruct all Authorised Users of the Licensed Work and Digital Maps that they are not permitted to use Digital Maps until a new Educational Copyright Licence is negotiated directly with Ordnance Survey.
  - Deactivate all registrations of Authorised Users at the offending Authorised Institution within 2 weeks.
  - The offending Authorised Institution may only be considered for access to the Licensed Work and Digital Maps under the terms of this Agreement (or any future replacement arrangements) after a period of three years.
  - Publicise within the higher and further education community that the offending Authorised Institution no longer has access to the Licensed Work and Digital Maps and that they may not receive the Licensed Work and Digital Maps from any other Authorised Institution - within 2 weeks.

## Appendix 4 - Paper and Electronic Publication Sizes

### 1. Paper Publication

The following table sets out the maximum permitted image sizes for circulation on paper. These apply to Ordnance Survey maps produced directly from Digimap and to maps created from Ordnance Survey data downloaded through Digimap.

| **Paper Publication type** | **Dataset** | **Maximum image Size per publication** | **Notes** |
| --- | --- | --- | --- |
| Conference papers | All dataset | 1250 cm^2^ (A3) | Any single image from Strategi®, Meridian™ 2 1:50,000 Scale Colour Raster, Land-Form PANORAMA™, 1:25,000 Scale Colour Raster, Land-Form PROFILE®, or 1:10,000 Scale Colour Raster must not exceed 625cm2 (A4) |
| Academic articles | All Datasets | 1250 cm^2^ (A3) | Any single image from Strategi®, Meridian™ 2 1:50,000 Scale Colour Raster, Land-Form PANORAMA™, 1:25,000 Scale Colour Raster, Land-Form PROFILE^®^, or 1:10,000 Scale Colour Raster must not exceed 625cm^2^ (A4) |
| Working papers and course notes | All datasets | No size limitations |  |
| Dissertations and theses | All datasets | No size limitations |  |
| Poster sessions | All datasets | No size limitations |  |
| Limited Internal Business Use* | All datasets | 1250 cm^2^ (A3) |  |

* Limited Internal Business Use allows incidental use of Ordnance Survey Mapping for display and promotional purposes, such as location maps published on an Authorised Institution's website and posters or fly leaflets to support business activities provided that no financial gain is made.

### 2. Electronic Publication

The following restrictions apply to the sizes of Ordnance Survey map extracts taken from the Digimap service and to images created from downloaded Ordnance Survey data when included for circulation in electronic form. The map image must not be created in real time for serving to the reader.

**2A. DVD, CD-Rom or floppy disk**

| **DVD, CD_ROM or floppy disk** | **Dataset** | **Maximum mapped area per image** | **Maximum mapped area per publication** |
| --- | --- | --- | --- |
| Conference Papers | All datasets | 200 cm^2^ at source scale | 1250 cm^2^ (A3) at source scale |
| Academic articles | All datasets | 200 cm^2^ at source scale | 1250 cm^2^ (A3) at source scale |
| Working papers and course notes | All datasets | 200 cm^2^ at source scale | No restriction |
| Dissertations and theses | All datasets | 200 cm^2^ at source scale | No restriction |
| Limited Internal Business Use | All datasets | No restriction | No restriction |

**2B. Intranet**

| **lntranet**** | **Dataset** | **Maximum mapped area per image** | **Maximum mapped area per publication** |
| --- | --- | --- | --- |
| Conference Papers | All datasets | 200 cm^2^ at source scale | 1250 cm^2^ (A3) at source scale |
| Academic articles | All datasets | 200 cm^2^ at source scale | 1250 cm^2^ (A3) at source scale |
| Working papers and course notes | All datasets | 200 cm^2^ at source scale | No restriction |
| Dissertations and theses | All datasets | 200 cm^2^ at source scale | No restriction |
| Limited internal Business Use | All datasets | No restriction | No restriction |

** NB - An Intranet is an internal network specific to a single Authorised Institution and only accessible to members of that Institution. This network is NOT connected to the public Internet. For Internet publication see table below.

**2C. Internet**

| **lnternet*** | **Dataset** | **Maximum mapped area per image** | **Maximum mapped area per publication** |
| --- | --- | --- | --- |
| Conference Papers | All datasets | 200 cm^2^ at source scale | 1250 cm^2^ (A3) at source scale |
| Academic articles | All datasets | 200 cm^2^ at source scale | 1250 cm^2^ (A3) at source scale |
| Working papers and course notes | All datasets | 200 cm^2^ at source scale | 1250 cm^2^ (A3) at source scale |
| Dissertations and theses | All datasets | 200 cm^2^ at source scale | 1250 cm^2^ (A3) at source scale |
| Limited internal Business Use | All datasets | No restriction | No restriction |

- Images may be displayed at any size on screen.
- More than one image may be included but no single image may be of a size greater than specified above.

**Source scale**

Source scale is the scale of the source information from which the map was digitised. You may include a map in your publication at any scale, so long as the map would be no larger than the specified size if it was mapped at the source scale for the product used. The table below shows the corresponding area on the ground for each of the maximum mapped areas specified above.

| **Product** | **Source Scale** | **Size at source scale** | **Ground area** |
| --- | --- | --- | --- |
| Strategi^®^ | 1:250,000 | 1250cm^2^ 200cm^2^ | 7812.5km^2^ 1250km^2^ |
| Meridian 2™ | 1:50,000 | 1250cm^2^ 200cm^2^ | 312.5km^2^ 50 km^2^ |
| 1:50 000 Scale Colour Raster | 1:50,000 | 1250cm^2^ 200cm^2^ | 312.5km^2^ 50 km^2^ |
| Land-Form PANORAMA™ | 1:50,000 | 1250cm^2^ 200cm^2^ | 312.5km^2^ 50 km^2^ |
| 1:25 000 Scale Colour Raster | 1:25,000 | 1250cm^2^ 200cm^2^ | 78.125 km2 12.5 km2 |
| Land-Form PROFILE^®^ | 1:10,000 | 1250cm^2^ 200cm^2^ | 12.5km^2^ 2km^2^ |
| 1:10 000 Scale Colour Raster | 1:10,000 | 1250cm^2^ 200cm^2^ | 12.5km^2^ 2km^2^ |
| OS MasterMap® | 1:1,250 | 1250cm2 200cm2 | 0.195 km2 (19.5 ha) 0.03125 km2 (3.125 ha) |
| Land-Line^®^: Urban tiles | 1:1,250 | 1250cm^2^ 200cm^2^ | 0.195 km^2^ (19.5 ha) 0.03125 km^2^ (3.125 ha) |
| Land-Line^®^: Rural tiles | 1:2,500 | 1250cm^2^ 200cm^2^ | 0.781 km^2^ (78.1 ha) 0.125 km^2^ (12 5 ha) |
| Land-Line^®^: Moorland tiles | 1:10,000 | 1250cm^2^ 200cm^2^ | 12.5km^2^ 2km^2^ |
