# Supplementary material for: Changes in the Distribution of Red Foxes (Vulpes vulpes) in Urban Areas in Great Britain: Findings and Limitations of a Media-Driven Nationwide Survey
Source: PLoS One. 2014 Jun 11;9(6):e99059. doi: 10.1371/journal.pone.0099059 (PMC4053368; doi:10.1371/journal.pone.0099059)
Supplement: Licence S2 — Ordinance Survey (OS) open data licence. (PDF) [file pone.0099059.s003.pdf]

This licence governs your access to and use of OS OpenData™ made available at https://www.ordnancesurvey.co.uk/opendatadownload/products.html and at http://data.ordnancesurvey.co.uk/ by The Secretary of State for Business, Innovation and Skills acting through Ordnance Survey (the 'Licensor' and 'Information Provider') under a delegation of authority.

It incorporates the Open Government Licence for public sector information (see below and http://www.nationalarchives.gov.uk/doc/open-government-licence/) which is varied by the following terms:

You must always use the following attribution statement to acknowledge the source of the information:

Contains Ordnance Survey data © Crown copyright and database right [year]

You must also use the following attribution statement where you use Code-Point® Open data:

Contains Royal Mail data © Royal Mail copyright and database right [year]
Contains National Statistics data © Crown copyright and database right [year]

The same attribution statements must be contained in any sub-licences of the Information that you grant, together with a requirement that any further sub-licences do the same.

OS OpenData™ is covered by either Crown Copyright, Crown Database Right, or has been licensed to the Crown.

Open Government Licence
for public sector information

delivered by The National Archives

You are encouraged to use and re-use the Information that is available under this licence freely and flexibly, with only a few conditions.

Using Information under this licence

Use of copyright and database right material expressly made available under this licence (the **Information**) indicates your acceptance of the terms and conditions below.

The Licensor grants you a worldwide, royalty-free, perpetual, non-exclusive licence to use the Information subject to the conditions below.

This licence does not affect your freedom under fair dealing or fair use or any other copyright or database right exceptions and limitations.

You are free to:

- copy, publish, distribute and transmit the Information;
- adapt the Information;
- exploit the Information commercially and non-commercially for example, by combining it with other Information, or by including it in your own product or application.

You must, where you do any of the above:

acknowledge the source of the Information by including any attribution statement specified by the Information Provider(s) and, where possible, provide a link to this licence;

If the Information Provider does not provide a specific attribution statement, or if you are using Information from several Information Providers and multiple attributions are not practical in your product or application, you may use the following:

Contains public sector information licensed under the Open Government Licence v2.0.

These are important conditions of this licence and if you fail to comply with them the rights granted to you under this licence, or any similar licence granted by the Licensor, will end automatically.

Exemptions

This licence does not cover:

- personal data in the Information;
- information that has neither been published nor disclosed under information access legislation (including the Freedom of Information Acts for the UK and Scotland) by or with the consent of the Information Provider;
- departmental or public sector organisation logos, crests and the Royal Arms except where they form an integral part of a document or dataset;
- military insignia;
- third party rights the Information Provider is not authorised to license;
- other intellectual property rights, including patents, trade marks, and design rights; and
- identity documents such as the British Passport.

Non-endorsement

This licence does not grant you any right to use the Information in a way that suggests any official status or that the Information Provider endorses you or your use of the Information.

Non warranty

The Information is licensed 'as is' and the Information Provider excludes all representations, warranties, obligations and liabilities in relation to the Information to the maximum extent permitted by law.

The Information Provider is not liable for any errors or omissions in the Information and shall not be liable for any loss, injury or damage of any kind caused by its use. The Information Provider does not guarantee the continued supply of the Information.

Governing Law

This licence is governed by the laws of the jurisdiction in which the Information Provider has its principal place of business, unless otherwise specified by the Information Provider.

Definitions

In this licence, the terms below have the following meanings:

'**Information**'

means information protected by copyright or by database right (for example, literary and artistic works, content, data and source code) offered for use under the terms of this licence.

‘**Information Provider**’

means the person or organisation providing the Information under this licence.

‘**Licensor**’

means any Information Provider who has the authority to offer Information under the terms of this licence. It includes the Controller of Her Majesty’s Stationery Office, who has the authority to offer Information subject to Crown copyright and Crown database rights, and Information subject to copyright and database rights which have been assigned to or acquired by the Crown, under the terms of this licence.

‘**Use**’

means doing any act which is restricted by copyright or database right, whether in the original medium or in any other medium, and includes without limitation distributing, copying, adapting, modifying as may be technically necessary to use it in a different mode or format.

‘**You**’

means the natural or legal person, or body of persons corporate or incorporate, acquiring rights under this licence.

About the Open Government Licence

The Controller of Her Majesty’s Stationery Office (**HMSO**) has developed this licence as a tool to enable Information Providers in the public sector to license the use and re-use of their Information under a common open licence. The Controller invites public sector bodies owning their own copyright and database rights to permit the use of their Information under this licence.

The Controller of HMSO has authority to license Information subject to copyright and database right owned by the Crown. The extent of the Controller’s offer to license this Information under the terms of this licence is set out on The National Archives website.

This is version 2.0 of the Open Government Licence. The Controller of HMSO may, from time to time, issue new versions of the Open Government Licence. If you are already using Information under a previous version of the Open Government Licence, the terms of that licence will continue to apply.

These terms are compatible with the Creative Commons Attribution License 4.0 and the Open Data Commons Attribution License, both of which license copyright and database rights. This means that when the Information is adapted and licensed under either of those licences, you automatically satisfy the conditions of the OGL when you comply with the other licence. The OGLv2.0 is Open Definition compliant.

Further context, best practice and guidance can be found in the UK Government Licensing Framework section on The National Archives website.

OGL
